# Fairness-aware supervised hierarchical contrastive semantic learning for sexual dimorphism analysis

**DOI:** 10.1093/bioinformatics/btag237

**Published:** 2026-07-07

**Authors:** Euiseong Ko, Sai Phani Parsa, Sai Chandra Kosaraju, Tesfaye B Mersha, Mingon Kang

**Affiliations:** Department of Biomedical Informatics and Data Science, Heersink School of Medicine, University of Alabama at Birmingham, Birmingham, AL 35294, United States; Department of Computer Science, University of Nevada, Las Vegas, NV 89154, United States; Department of Computer Science, California State Polytechnic University, Pomona, CA 91768, United States; Precision Pulmonary Medicine Research Program, Department of Medicine, Division of Pulmonary, Critical Care, and Sleep Medicine, Indiana University School of Medicine, Indianapolis, IN, USA; Department of Computer Science, University of Nevada, Las Vegas, NV 89154, United States

## Abstract

**Motivation:**

Sexual dimorphism is a fundamental biological determinant driving systematic differences in disease susceptibility, progression, and clinical outcomes. However, current sex-combined AI-based genomic models often exhibit algorithmic bias and fail to capture these sex-specific mechanisms, creating a critical barrier to unbiased precision medicine. Ensuring fairness in the context of sexual dimorphism requires understanding and addressing the distinct biological mechanisms functioning in each sex, rather than focusing solely on equalizing predictive performance.

**Results:**

We propose a fairness-aware supervised hierarchical contrastive learning approach, called FairHICON, to discover unbiased sex-common and sex-specific predictive features. Evaluations on cancer and asthma transcriptomic datasets demonstrate that FairHICON significantly outperforms state-of-the-art benchmarks, improving predictive performance by up to 9% while effectively reducing the performance gap between male and female sexes. Furthermore, prognostic validation confirms that the identified sex-specific pathways stratify patient survival significantly better within their corresponding sex groups. This validates FairHICON to elucidate the molecular heterogeneity of sexual dimorphism, advancing inclusive precision medicine.

**Availability and implementation:**

The source code and data is available at https://github.com/datax-lab/FairHICON.

## 1 Introduction

Biological sex significantly influences human physiology, leading to substantial differences in disease susceptibility, progression, and clinical outcomes (Ober *et al.* 2008, Kim *et al.* 2018, Zheng *et al.* 2019, Jun *et al.* 2021). For instance, asthma prevalence shifts during development; it is more common in boys during childhood, but affects women more frequently and severely after puberty, indicating a specific interaction between sex hormones and immune function (Postma 2007). In oncology, men generally show higher incidence rates and lower survival probabilities than women for various cancers, including those of the bladder, colon, liver, and brain (Cook *et al.* 2011, Rubin *et al.* 2020, Siegel *et al.* 2021). These phenotypic observations stem from distinct molecular mechanisms, characterized by divergent regulatory networks and pathogenic pathways that differ between males and females (Lopes-Ramos *et al.* 2018, 2020, Oliva *et al.* 2020, Hartman *et al.* 2021).

Analytical methodologies have advanced in elucidating the molecular mechanisms underlying sexual dimorphism. Conventional statistical approaches, such as differential expression analysis, established the foundation for identifying genes associated with sex-specific traits (Gautam *et al.* 2019, Bourquard *et al.* 2023). Mostly, these studies have relied on independent analyzes that examine male and female cohorts separately to isolate sex-specific associations. The field has shifted toward advanced deep learning models to capture non-linear relationships in high-dimensional genomic data (Hases *et al.* 2021, Ko *et al.* 2024). For instance, the Sex-specific and Pathway-based Interpretable Neural network (SPIN) incorporates biological pathway information to detect sex-specific molecular features in cancer and asthma (Ko *et al.* 2024). These advances demonstrate the increasing capacity of AI to characterize the genomic basis of sex differences.

Despite these methodological advances, the application of AI to translational genomics faces significant challenges in terms of algorithmic fairness. Most deep learning models are susceptible to reinforcing inherent biases from data, particularly when training data contain substantial imbalances between male and female populations (Ober *et al.* 2008). These models frequently exhibit skewed bias toward the majority group or rely on misleading statistical associations between sex and disease, rather than uncovering stable, causal molecular mechanisms. Such biases manifest as clear differences in performance, providing stronger predictive power for one sex while reducing how well the model generalizes to the other.

In the context of sexual dimorphism, it is critical to distinguish between mitigating *algorithmic imbalance* and modeling *true biological dimorphism*. Algorithmic imbalance occurs when a model leverages spurious statistical correlations driven by imbalanced training data using sex as a shortcut for disease prediction, which manifests as predictive disparity. In contrast, true biological dimorphism acknowledges that the underlying ground-truth pathogenic mechanisms may fundamentally differ between sexes. Forcing a singular representation collapses this dimorphism, inherently causing algorithmic bias against the biologically distinct minority.

To address this critical gap, we propose a novel approach, Fairness-aware Supervised Hierarchical Contrastive Semantic Learning (FairHICON), to mitigate sex bias while uncovering the discovery of unbiased sex-common/-specific genomic mechanisms. FairHICON introduces a novel two-level hierarchical supervised contrastive loss designed to identify and characterize the specific molecular determinants present in each sex group. This objective methodically refines the structure of the latent space by using phenotypic labels to maintain clear separation between classes, while simultaneously exploiting sex attributes to enforce disentanglement across subgroups. By further integrating adaptive prototype weighting with pathway architectures informed by biological prior knowledge, FairHICON ensures that the learned representations remain both statistically fair and biologically interpretable for both sexes. The main contributions of FairHICON are (i) a novel fairness-aware approach integrating supervised hierarchical contrastive learning with biologically informed architectures to ensure unbiased representation learning; (ii) superior predictive performance that effectively closes the accuracy gap between male and female cohorts compared to state-of-the-art benchmarks; and (iii) fair and interpretable discovery, enabling the identification of clinically relevant sex-common and sex-specific biological risk factors for both sexes.

## 2 Materials and methods

We introduce FairHICON that mitigates sex disparities in predictive performance and model interpretation by learning unbiased sex-specific and sex-common transcriptomic mechanisms. The following sections outline the theoretical basis of contrastive learning for the analysis of sexual dimorphism, describe the network architecture and the hierarchical objective function of FairHICON, and present the training strategy used to enhance both stability and interpretability.

### 2.1 Contrastive learning for sexual dimorphism

Contrastive learning is a representation learning paradigm designed to optimize the geometry of a latent feature space. The fundamental principle of contrastive learning is to pull semantically similar samples (positive pairs) closer together while pushing dissimilar samples (negative pairs) apart. This learning process organizes data vectors based on their intrinsic relationships, creating a structured embedding space where the spatial distance directly corresponds to semantic similarity.

In the context of sexual dimorphism analysis, the contrastive learning provides a powerful mechanism for feature disentanglement. A typical application could define similarity based on a straightforward pairing of clinical outcome and sex (e.g. treating male-disease and female-disease as completely separate categories). However, this combinatorial method has a significant drawback. By treating these subgroups as strictly negative pairs, the standard contrastive objective forces the model to separate them in the latent space. Although this rigorous separation highlights sex-specific distinctions, it inadvertently suppresses sex-common biological mechanisms that are fundamentally shared throughout the disease regardless of sex. As a result, a straightforward combinatorial method cannot simultaneously preserve these conserved signals and separate sex-specific heterogeneity at the same time, making it necessary to adopt a hierarchical strategy that can balance these opposing objectives.

### 2.2 The FairHICON architecture

**Figure 1:**
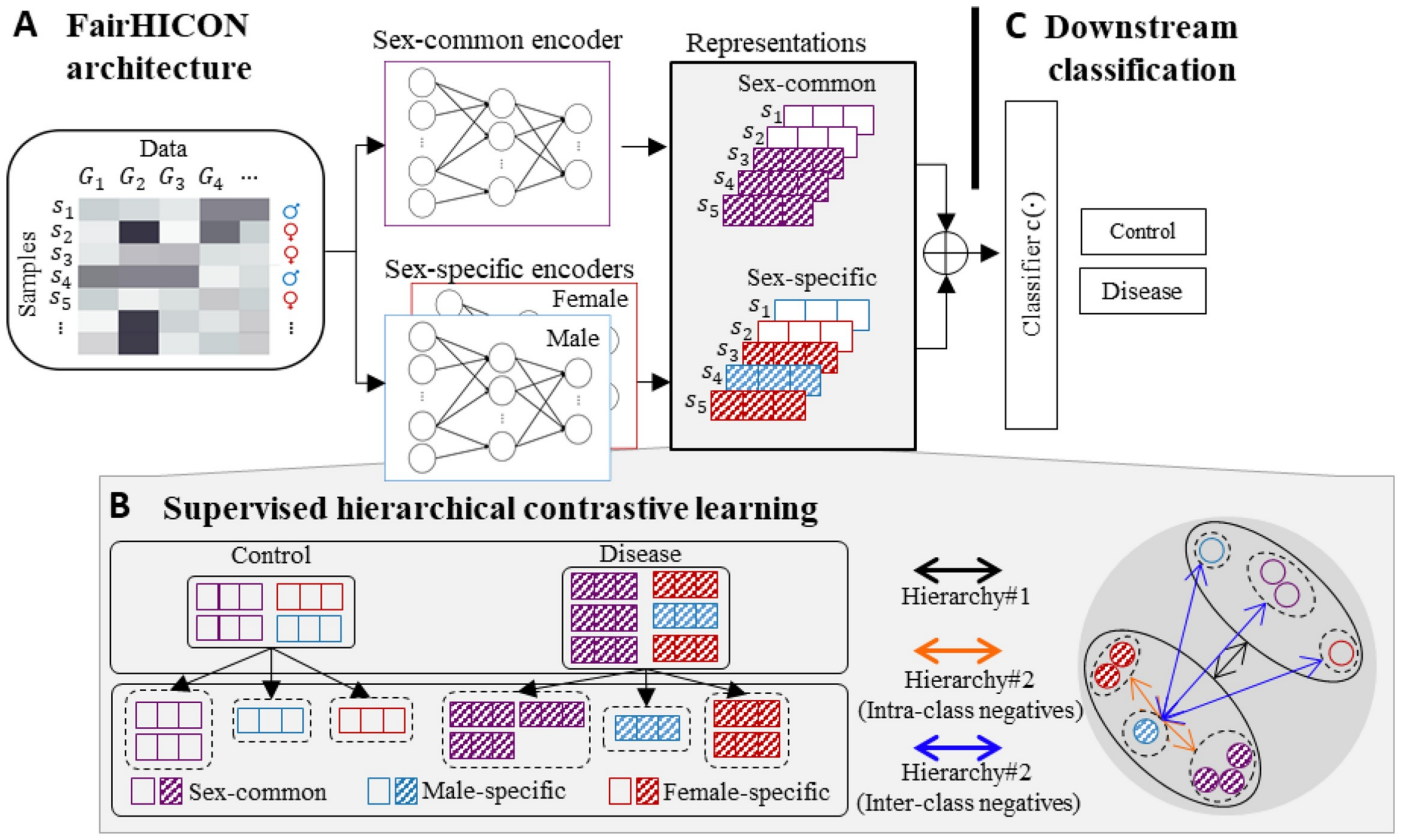
Overview of FairHICON. The approach takes gene expression data and sex attribute as input, processing them through a Siamese architecture. The sex-common backbone model extracts features shared across groups, while sex-specific models (male and female) capture sex-specific patterns. These representations are optimized via supervised hierarchical contrastive learning (bottom panel), which organizes the latent space representations into two levels: Level 1 Hierarchy (Hierarchy#1) separates samples based on the primary clinical outcome (control versus disease), while Level 2 Hierarchy (Hierarchy#2) refines the feature space using hard negatives to distinguish subgroup representations between different classes, and soft negatives to disentangle subgroup features within same class. These representations are fused (⊕) to perform the downstream tasks, specifically cancer survival prediction (classifying Long-Term Survivors, LTS, versus Non-LTS) and asthma risk score prediction (stratifying patients). (A) Architecture overview showing the sex-common and sex-specific encoders to produce corresponding representations; (B) Schematic of the Level 1 and Level 2 hierarchical contrastive learning objectives; (C) Downstream clinical task integration for survival and risk prediction.

FairHICON uses a multi-branch Siamese architecture designed to explicitly disentangle sex-specific mechanisms from shared disease drivers (Fig. 1). Given the labeled dataset, let $D={\left\{(x_{i},y_{i},s_{i})\right\}}_{i=1}^{N}$, where $x_{i}$ represents the gene expression profile (e.g. RNA-seq), $y_{i}\in \{0,1\}$ denotes the clinical outcome, and $s_{i}\in \{M,F\}$ represents the sex attribute.

The architecture consists of three distinct encoders: a sex-common encoder ($E_{C}$) to capture sex-common features, and two sex-specific encoders ($E_{M}$ and $E_{F}$) to capture mechanisms unique to males and females. To ensure biological interpretability, each encoder is adapted using the Pathway-Associated Sparse Deep Neural Network (PASNet) architecture (Hao *et al.* 2018). Unlike standard fully connected networks, PASNet enforces sparse connectivity constrained by prior biological knowledge (e.g. KEGG pathway annotations). This design ensures that the internal topology of the network mirrors hierarchical biological systems, allowing the direct attribution of predictive signals to specific molecular pathways. The information flows through three specific transformation stages:

The gene-to-pathway layer constitutes the input stage, where $d_{g}$ nodes correspond to individual genes mapping to $d_{p}$ nodes representing biological pathways. Following the PASNet structure, the weight matrix $W^{\left(1\right)}$ is constrained by a binary mask $M\in {\left\{0,1\right\}}^{d_{g}\times d_{p}}$, where $M_{jk}=1$ if and only if gene *j* is annotated to pathway *k*. The forward propagation for the pathway activation vector *h* is computed as:


(1) $$h=\sigma ({\left(W^{\left(1\right)}⊙M\right)}^{T}x+b^{\left(1\right)}),$$

where ⊙ denotes the element-wise Hadamard product, which strictly zeroes out weights corresponding to non-existent biological relationships.

Subsequently, the pathway-to-representation layer aggregates the pathway activations *h* into a lower-dimensional latent embedding vector $z_{r}\in R$ via a fully connected layer. This layer synthesizes the activity of disparate biological processes into a compact representation:


(2) $$z_{r}=E(x)=\sigma (W^{\left(2\right)}h+b^{\left(2\right)})$$

Finally, during the contrastive pre-training phase, a projection head H(·) maps the representation into the metric space where the loss is calculated. This head consists of a two-layer MLP ($z_{\mathrm{proj}}=W^{\left(4\right)}\sigma (W^{\left(3\right)}z_{r})$). This design decouples representation learning from contrastive optimization, allowing the encoder to retain the information necessary for downstream tasks, while the projection head absorbs the geometric distortions induced by contrastive loss (Chen *et al.* 2020).

For every patient sample *i*, the approach generates a dual-view representation comprising a sex-common view, denoted as $z_{i}^{C}=\text{Norm}(H(E_{C}(x_{i})))$, and a sex-specific view, denoted as $z_{i}^{S}=\text{Norm}(H(E_{s_{i}}(x_{i})))$. Here, H(·) is the projection head and Norm(·) denotes $L_{2}$ normalization. These views are aggregated into an augmented batch Z of size 2*N*, indexed by k∈{1,…,2N}. Each embedding in the batch is explicitly associated with a semantic triplet $\left(y_{k},s_{k},t_{k}\right)$, where $t_{k}\in \{\text{common},\text{male}-/\text{female-specific}\}$ denotes the encoder origin.

### 2.3 Supervised hierarchical contrastive semantic loss

We propose the Supervised Hierarchical Contrastive Semantic Loss (SHCSL) that learns discriminative representations of clinical outcomes and invariant to sex-based biases. This objective function is formulated in the two hierarchical levels of inter-class separability and intra-class disentanglement.

The total objective function aggregates the hierarchical level losses (*h*1 and *h*2) which are computed on both the projection space and the representation space. The total loss is defined as:


(3) $$L_{\mathrm{SHCSL}}=L_{\mathrm{total}}^{h1}+\lambda (t)\cdot L_{\mathrm{total}}^{h2}.$$

The hierarchical losses consider four semantic relationship between an anchor sample *u* and any other sample *v*, defined as:

- Intra-Class, Intra-Attribute ($S_{\mathrm{ICIA}}$) is the similarity between an anchor *u* and samples belonging to the exact same subgroup, defined as $S_{\mathrm{ICIA}}(u)=\{v\in Z\setminus u|y_{v}=y_{u}\;\wedge \;s_{v}=s_{u}\;\wedge \;t_{v}=t_{u}\}$.
- Intra-Class, Inter-Attribute ($S_{\mathrm{ICEA}}$) is the similarity between an anchor *u* and samples sharing the clinical label but differing in subgroup (different sex or encoder origin), defined as $S_{\mathrm{ICEA}}(u)=\{v\in Z\setminus \{u\}|y_{v}=y_{u}\;\wedge \;(s_{v}\neq s_{u}\vee t_{v}\neq t_{u})\}$.
- Inter-Class, Intra-Attribute ($S_{\mathrm{ECIA}}$) is the similarity between an anchor *u* and samples with different clinical labels but sharing sensitive attributes, defined as $S_{\mathrm{ECIA}}(u)=\{v\in Z\setminus \{u\}|y_{v}\neq y_{u}\;\wedge \;s_{v}=s_{u}\}$.
- Inter-Class, Inter-Attribute ($S_{\mathrm{ECEA}}$) is the similarity between an anchor *u* and samples differing in both label and attributes, defined as $S_{\mathrm{ECEA}}(u)=\{v\in Z\setminus \{u\}|y_{v}\neq y_{u}\;\wedge \;s_{v}\neq s_{u}\}$.

The first level hierarchy maximizes global predictive accuracy by clustering samples based strictly on the clinical outcome. The positive set ($P_{H1}$) includes all samples sharing the same clinical label *y*, regardless of sex or encoder origin, defined as $P_{H1}(u)=S_{\mathrm{ICIA}}(u)\cup S_{\mathrm{ICEA}}(u)$. We use standard uniform weighting ($w_{u,a}=1$) to learn robust decision boundaries. To mitigate the impact of group imbalance, the loss uses group-wise normalization: the loss is calculated independently for each unique subgroup and then averaged, ensuring that minority groups contribute equitably to the gradient (Park *et al.* 2022):


(4) $$L_{g}^{h1}=\frac{1}{\left|g\right|}\sum_{i\in g}\frac{-1}{\left|P(i)\right|}\sum_{p\in P(i)}\;\text{log}\;\frac{\;\text{exp}(z_{i}\cdot z_{p}/\tau_{1})}{\sum_{a\in A(i)}\;\text{exp}\;(z_{i}\cdot z_{a}/\tau_{1})},$$

where $\tau_{1}$ is a standard temperature parameter.

The second level hierarchy promotes fairness by disentangling subgroups within the same disease class. The positive set ($P_{H2}$) is restricted strictly to samples from the same semantic subgroup, such that $P_{H2}(u)=S_{\mathrm{ICIA}}(u)$. To effectively structure the latent space, we categorize negative samples into two structural types with distinct optimization goals. Intra-class negatives ($N_{\mathrm{intra}}$) are samples sharing the clinical label $y_{u}$ but belonging to a different semantic subgroup ($N_{\mathrm{intra}}(u)=S_{\mathrm{ICEA}}(u)$); these samples are structurally proximal due to $H_{1}$ but require active penalization to prevent the merging of sex-specific manifolds. Inter-class negatives ($N_{\mathrm{inter}}$) are samples belonging to the opposing clinical class ($N_{\mathrm{inter}}(u)=S_{\mathrm{ECIA}}(u)\cup S_{\mathrm{ECEA}}(u)$), representing the fundamental decision boundary. To establish a stable reference for each semantic cluster, we compute a group prototype $c_{g}$ for every unique subgroup g∈G. This prototype is defined as the $L_{2}$-normalized centroid of all embeddings belonging to that subgroup:


(5) $$c_{g}=\text{Norm}\left(\frac{1}{\left|I_{g}\right|}\sum_{i\in I_{g}}z_{i}\right),$$

where $I_{g}$ denotes the set of indices for samples belonging to subgroup *g*, and Norm(·) applies $L_{2}$ normalization.

We then use an adaptive weighting mechanism, $w_{u,a}$, to penalize negatives that violate the subgroup structure relative to the anchor’s group prototype $c_{g(u)}$:


(6) $$w_{u,a}=\{\begin{array}{c} \;\text{exp}\;\left(\alpha_{\mathrm{type}}\cdot (z_{u}\cdot z_{a}-z_{u}\cdot c_{g(u)})\right)\;\text{if}\;z_{u}\cdot z_{a}>z_{u}\cdot c_{g(u)}\\ 1\;\;\;\;\text{otherwise} \end{array}$$

We assign distinct scaling factors, $\alpha_{\mathrm{intra}}$ and $\alpha_{\mathrm{inter}}$, to the respective negative sets. We set $\alpha_{\mathrm{intra}}<\alpha_{\mathrm{inter}}$ to prioritize the stability of the global decision boundary (separating disease versus control) while sufficiently penalizing intra-class overlap to enforce fine-grained, sex-specific disentanglement. To ensure numerical stability and adherence to column width constraints, we define the weighted denominator term $D_{\mathrm{weighted}}(i)$ separately: The weighted contrastive loss is defined as:


(7) $$L_{g}^{h2}=\frac{1}{\left|g\right|}\sum_{i\in g}\frac{-1}{\left|P(i)\right|}\sum_{p\in P(i)}\;\text{log}\;\frac{\;\text{exp}(z_{i}\cdot z_{p}/\tau_{2})}{D_{\mathrm{weighted}}(i)},$$

where the weighted denominator $D_{\mathrm{weighted}}(i)$ aggregates contributions from positive pairs and weighted negative samples. Let N(i) be the set of negative indices for the anchor *i* (i.e. N(i) = A(i)∖P(i)). Then:


(8) $$D_{\mathrm{weighted}}(i)=\sum_{p^{\prime }\in P(i)}\;\text{exp}\;(z_{i}\cdot z_{p^{\prime }}/\tau_{2})+\sum_{n\in N(i)}w_{i,n}\cdot \;\text{exp}(z_{i}\cdot z_{n}/\tau_{2}),$$

where $\tau_{2}$ is the temperature parameter for the second hierarchy. The weighted denominator, $D_{\mathrm{weighted}}(i)$, controls the latent space geometry by adaptively penalizing intruding negative samples. Unlike standard contrastive denominators that treat all negative samples uniformly and risk destabilizing global class boundaries, this formulation incorporates the weight $w_{u,a}$ [Equation (6)] to scale the repulsive force against any negative sample entering the anchor’s semantic cluster (i.e. when $z_{u}\cdot z_{a}>z_{u}\cdot c_{g(u)}$). Furthermore, it applies distinct scaling factors based on the sample’s structural type: $\alpha_{\mathrm{intra}}$ strongly penalizes overlapping intra-class negatives to drive fine-grained, sex-specific disentanglement, while $\alpha_{\mathrm{inter}}$ scales the repulsion against inter-class negatives to preserve the overall clinical decision boundary. This targeted weighting resolves subgroup overlap without compromising the primary predictive manifolds.

To ensure the structural integrity of the learned features, the component losses for each hierarchy are calculated as a weighted sum of the loss on the projection and representation spaces, controlled by a balance parameter α:


(9) $$L_{\mathrm{total}}^{h}=L^{h}(z^{\mathrm{proj}})+\alpha \cdot L^{h}(z^{\mathrm{rep}}),$$

where the representations $z^{\mathrm{rep}}$ are $L_{2}$-normalized specifically for this loss calculation.

### 2.4 Training strategy

We use a two-phase curriculum learning strategy to stabilize the optimization of the hierarchical objective (Wang *et al.* 2021). In Phase 1, the network was optimized exclusively using the first-level hierarchy loss ($L_{\mathrm{total}}^{h1}$) for the first 500 epoch to establish robust disease classification boundaries between disease and control groups without the constraint of subgroup disentanglement. In Phase 2, the second-level hierarchy loss ($L_{\mathrm{total}}^{h2}$) was introduced to enforce fairness and disentanglement. To prevent destabilization of the learned features, the weight λ(epoch) is applied starting at epoch $E_{\mathrm{start}}$, linearly increasing from zero to a target value $\lambda_{\mathrm{target}}$ over a warm-up period of $E_{\mathrm{warmup}}$ epochs. With $E_{\mathrm{start}}=501$ and $E_{\mathrm{warmup}}=500$, the adaptive weighting function is formally defined as:


(10) $$\lambda (\mathrm{epoch})=\{\begin{array}{c} 0\;\;\;\;\text{if}\;\mathrm{epoch}<E_{\mathrm{start}}\\ \lambda_{\mathrm{target}}\cdot \min\left(1,\frac{\mathrm{epoch}-E_{\mathrm{start}}}{E_{\mathrm{warmup}}}\right)\;\text{if}\;\mathrm{epoch}\geq E_{\mathrm{start}} \end{array}$$

After epoch 1000, λ(epoch) remains constant at $\lambda_{\mathrm{target}}$, allowing the model to gradually refine the latent space and separate sex-specific manifolds within the established class clusters. Model selection relies on early stopping determined by the silhouette score of the validation set representations. While validation loss or Area Under the ROC Curve (AUROC) are standard metrics for classification tasks, they are sub-optimal for representation learning; AUROC primarily measures linear separability, and contrastive loss can fluctuate without reflecting true structural improvements. Since FairHICON aims to achieve strict geometric disentanglement of sex-specific and sex-common subgroups, the silhouette score is utilized to directly quantify cluster cohesion and inter-cluster separation. This ensures the model state is saved precisely when the subgroups reach optimal structural integrity. Furthermore, automated hyperparameter optimization (e.g. Optuna) across a wide distribution of values ($\tau_{1}$, $\tau_{2}$, α, λ) demonstrated that the curriculum learning warm-up schedule stabilizes the latent space geometry across diverse hyperparameter initializations.

### 2.5 Downstream binary classification

Following the contrastive learning, the projection heads are discarded, and the BINN encoders ($E_{C},E_{M},E_{F}$) are frozen to serve as feature extractors. For a patient *i* with sex $s_{i}$, we generate a fused representation $v_{i}$ by concatenating the unnormalized features from the sex-common and the corresponding sex-specific encoder:


(11) $$v_{i}=E_{C}(x_{i})⊕E_{s_{i}}(x_{i})$$

This joint vector $v_{i}\in R$ encapsulates both the common biological context and the specific characteristics of the sexual dimorphism. A classifier (e.g. a neural network) is subsequently trained on $v_{i}$ to predict the binary clinical outcome $y_{i}$. The classifier is optimized using standard Binary Cross-Entropy (BCE) loss.

### 2.6 Feature attribution and interpretation

To identify the specific sex-differential features contributing to the model’s predictions, we leverage the interpretable BINN architecture. We compute importance scores for both biological pathways and individual genes by calculating the gradient of the model’s predictive output with respect to the intermediate pathway layer activations and the input gene expression layer, respectively.

For a given patient sample *i*, let ${\hat{y}}_{i}$ denote the predicted clinical outcome (risk score). We compute the gradient vectors for the gene layer ($g_{i}^{\mathrm{gene}}$) and the pathway layer ($g_{i}^{\mathrm{path}}$) via backpropagation: $g_{i}^{\mathrm{gene}}=\frac{\partial {\hat{y}}_{i}}{\partial x_{i}},\;g_{i}^{\mathrm{path}}=\frac{\partial {\hat{y}}_{i}}{\partial h_{i}}$, where $x_{i}$ represents the input gene expression vector and $h_{i}$ represents the hidden state vector of the pathway layer. The Importance Score, *S*, for a specific feature (gene *j* or pathway *k*) is defined as the absolute value of its corresponding gradient component: $S(\mathrm{Gen}e_{j})=|g_{ij}^{\mathrm{gene}}|,\;S(\mathrm{Pathwa}y_{k})=|g_{ik}^{\mathrm{path}}|$ We compute these scores separately for the sex-common ($E_{C}$) and sex-specific ($E_{M},E_{F}$) encoders to explicitly distinguish between sex-conserved and sex-dimorphic mechanisms.

To identify robust population-level predictors, statistical testing is performed on the distribution of importance scores across the patient cohort. For each feature, a one-sample t-test is conducted to determine whether the mean importance score differs significantly from zero ($H_{0}:\mu_{S}=0$). To control for the false discovery rate (FDR) inherent in high-dimensional genomic data, *P*-values are adjusted using the Benjamini-Hochberg (BH) procedure. Features with an FDR-adjusted *P*-value < .01 are considered statistically significant and prioritized for further biological analysis.

## 3 Results

We evaluated FairHICON across four independent transcriptomic datasets to assess its ability to predict clinical outcomes accurately and to elucidate the underlying biological mechanisms of sexual dimorphism. We used RNA-Seq gene expression profiles to address two distinct clinical tasks: (i) cancer survival prediction and (ii) asthma risk stratification. For survival prediction, we obtained data from The Cancer Genome Atlas (TCGA), focusing on Liver Hepatocellular Carcinoma (LIHC), Lung Adenocarcinoma (LUAD) and Lower Grade Brain Glioma (LGG). Long-term survival (LTS) was defined as patients surviving ≥3 years, while those deceased within 3 years were categorized as non-LTS. Patients alive with survival time ≤3 years were treated as censored data and excluded from the analysis. For risk score prediction, we utilized an asthma dataset (nasal epithelium, GSE240567) obtained from the Gene Expression Omnibus (GEO). A summary of the characteristics of the dataset is provided in Table 1.

**Table 1:** Summary of TCGA and GEO datasets used in this study.

Dataset	Gene #	Patient # (neg/pos)	Male #/female #
LIHC	4781	197 (104/93)	129/68
LUAD	4786	272 (137/135)	128/144
LGG	4789	246 (79/167)	141/105
GSE240567	4500	536 (283/253)	206/330

To mitigate data redundancy and ensure high-quality inputs, we performed rigorous pre-processing by removing duplicate patient records, excluding samples missing critical target variables, and filtering out genes with missing values in over 80% of samples. The datasets were subsequently divided into training (64%), validation (16%), and testing (20%) sets via sex-stratified random sampling. To ensure result stability, experiments were replicated ten times; for each independent run, features were standardized by scaling the validation and testing sets using the mean and standard deviation derived strictly from the training set.

To ensure that performance differences are strictly attributable to the learning objective rather than model size, all evaluated baselines and FairHICON were constrained to comparable parameter counts and identical backbone capacities. Specifically, all models utilize the same underlying PASNet architecture with matched hidden layer dimensions. While FairHICON uses additional projection heads during the contrastive training phase to calculate the hierarchical loss, these components do not artificially inflate the capacity of the final predictive model. Consequently, the downstream classifiers used to generate the comparative ROC curves operate with matched architectural complexity.

### 3.1 Comparative evaluation of predictive performance and fairness

To evaluate the predictive performance and algorithmic fairness of FairHICON, we benchmarked it against three recent methods: contrastive learning with an MLP (CL-MLP) or XGBoost (CL-XGBoost), and the domain-specific SPIN model. As summarized in Table 2, FairHICON consistently achieved the highest overall and sex-stratified predictive accuracy (AUROC and AUPRC) across all evaluated datasets, while simultaneously minimizing bias metrics (Demographic Parity Difference and Equalized Odds Difference). This demonstrates that our hierarchical objective effectively mitigates algorithmic imbalance without sacrificing predictive utility.

**Table 2:** Comparative evaluation of predictive performance and fairness metrics across cancer (TCGA) and asthma (GSE240567) datasets.^a^

Dataset	Method	**Area under ROC curve (** ↑ **)**			**Area under PR curve (** ↑ **)**			**Fairness metrics (** ↓ **)**	
		Overall	**Sex-stratified**		Overall	**Sex-stratified**		DPD	EOD
			Male	Female		Male	Female		
**LGG**	CL-MLP	0.858 ± 0.040	0.933 ± 0.032	0.746 ± 0.096	0.844 ± 0.066	0.931 ± 0.037	0.776 ± 0.101	0.068 ± 0.030	0.206 ± 0.095
	CL-XGBoost	0.823 ± 0.066	0.882 ± 0.067	0.731 ± 0.099	0.853 ± 0.047	0.896 ± 0.058	0.810 ± 0.066	0.061 ± 0.035	0.230 ± 0.120
	SPIN	0.835 ± 0.062	0.894 ± 0.056	0.721 ± 0.133	0.811 ± 0.083	0.862 ± 0.076	0.757 ± 0.144	0.103 ± 0.024	0.283 ± 0.106
	**FairHICON**	**0.892** ± 0.047**	**0.940** ± **0.044**	**0.807** ± 0.085**	**0.893** ± 0.051**	**0.938** ± **0.051**	**0.839** ± **0.082**	0.070 ± 0.024	**0.188** ± **0.059**
**LIHC**	CL-MLP	0.662 ± 0.039	0.692 ± 0.054	0.581 ± 0.078	0.646 ± 0.053	0.703 ± 0.068	0.549 ± 0.083	0.085 ± 0.027	0.161 ± 0.034
	CL-XGBoost	0.650 ± 0.096	0.682 ± 0.130	0.583 ± 0.147	0.634 ± 0.098	0.680 ± 0.124	0.557 ± 0.155	0.121 ± 0.067	0.252 ± 0.122
	SPIN	0.620 ± 0.043	0.660 ± 0.067	0.526 ± 0.144	0.608 ± 0.065	0.643 ± 0.089	0.576 ± 0.118	**0.076** ± **0.045**	**0.156** ± **0.098**
	**FairHICON**	**0.722** ± 0.056*	**0.762** ± **0.057**	**0.636** ± **0.140**	**0.692** ± **0.089**	**0.742** ± **0.081**	**0.634** ± **0.158**	0.081 ± 0.043	0.175 ± 0.053
**LUAD**	CL-MLP	0.624 ± 0.051	**0.658** ± **0.103**	0.593 ± 0.068	**0.628** ± **0.062**	**0.645** ± **0.123**	0.636 ± 0.075	**0.037** ± **0.013**	**0.077** ± **0.025**
	CL-XGBoost	0.582 ± 0.053	0.581 ± 0.133	0.582 ± 0.075	0.585 ± 0.050	0.576 ± 0.139	0.610 ± 0.054	0.062 ± 0.044	0.116 ± 0.063
	SPIN	0.546 ± 0.043	0.604 ± 0.073	0.487 ± 0.079	0.541 ± 0.036	0.589 ± 0.097	0.539 ± 0.058	0.045 ± 0.040	0.082 ± 0.048
	**FairHICON**	**0.638** ± **0.079**	0.644 ± 0.106	**0.628** ± **0.113**	0.626 ± 0.079	0.607 ± 0.113	**0.657** ± **0.107**	0.058 ± 0.034	0.116 ± 0.047
**GSE240567**	CL-MLP	0.698 ± 0.066	0.697 ± 0.090	0.699 ± 0.061	0.697 ± 0.068	0.696 ± 0.073	0.711 ± 0.079	0.047 ± 0.015	0.075 ± 0.018
	CL-XGBoost	0.692 ± 0.066	0.685 ± 0.093	0.694 ± 0.072	0.691 ± 0.080	0.693 ± 0.085	0.694 ± 0.097	0.072 ± 0.043	0.134 ± 0.068
	SPIN	0.681 ± 0.033	0.677 ± 0.059	0.685 ± 0.047	0.700 ± 0.041	0.689 ± 0.079	0.714 ± 0.041	0.038 ± 0.010	0.069 ± 0.015
	**FairHICON**	**0.734** ± 0.044*	**0.735** ± 0.089**	**0.734** ± **0.032**	**0.737** ± 0.052**	**0.719** ± **0.101**	**0.757** ± 0.040*	**0.036** ± **0.012**	**0.066** ± **0.017**

a Values are reported as mean ± standard deviation. **Bold** indicates the best performance, and underlined indicates the second-best performance. Statistical significance of FairHICON compared to the best benchmark method is denoted by asterisks: *P<.05 and **P<.01. Arrows indicate optimization direction (↑ higher is better; ↓ lower is better).

To further evaluate the specific added value of our hierarchical disentanglement, we compared the predictive performance of FairHICON against a naive sex-stratified PASNet baseline (Table 1, available as supplementary data at *Bioinformatics* online). FairHICON significantly outperformed the stratified baseline by mitigating the severe data fragmentation and predictive instability commonly observed in harder-to-predict subgroups (e.g. FairHICON achieved a female-only LIHC AUROC of 0.722±0.056, compared to the baseline’s 0.521±0.138). These results confirm that our joint representation learning framework maximizes sample efficiency and effectively isolates sex-specific features without the representational penalties associated with dataset partitioning.

FairHICON mitigates algorithmic bias and maintains high predictive accuracy by enforcing strict geometric disentanglement within the latent space. Specifically, the Level 1 hierarchy maximizes global predictive accuracy by clustering samples into disease versus control groups, whereas the Level 2 hierarchy mathematically enforces fairness by disentangling these primary clusters into six distinct semantic subgroups (sex-common, male-specific, and female-specific). A detailed formal mapping of how this geometric optimization mathematically minimizes the reported post-hoc fairness metrics (Demographic Parity Difference and Equalized Odds Difference) is provided in Supplementary Note 1, available as supplementary data at *Bioinformatics* online.

### 3.2 Latent space disentanglement and structure

A key advantage of FairHICON is its ability to learn representations that are simultaneously accurate and structured. This is facilitated by the two-level hierarchical contrastive objective. The first level ($h_{1}$) prioritizes inter-class separability based on phenotype (clinical outcome), while the second level ($h_{2}$) facilitates intra-class disentanglement based on sex attributes and representation type.

**Figure 2:**
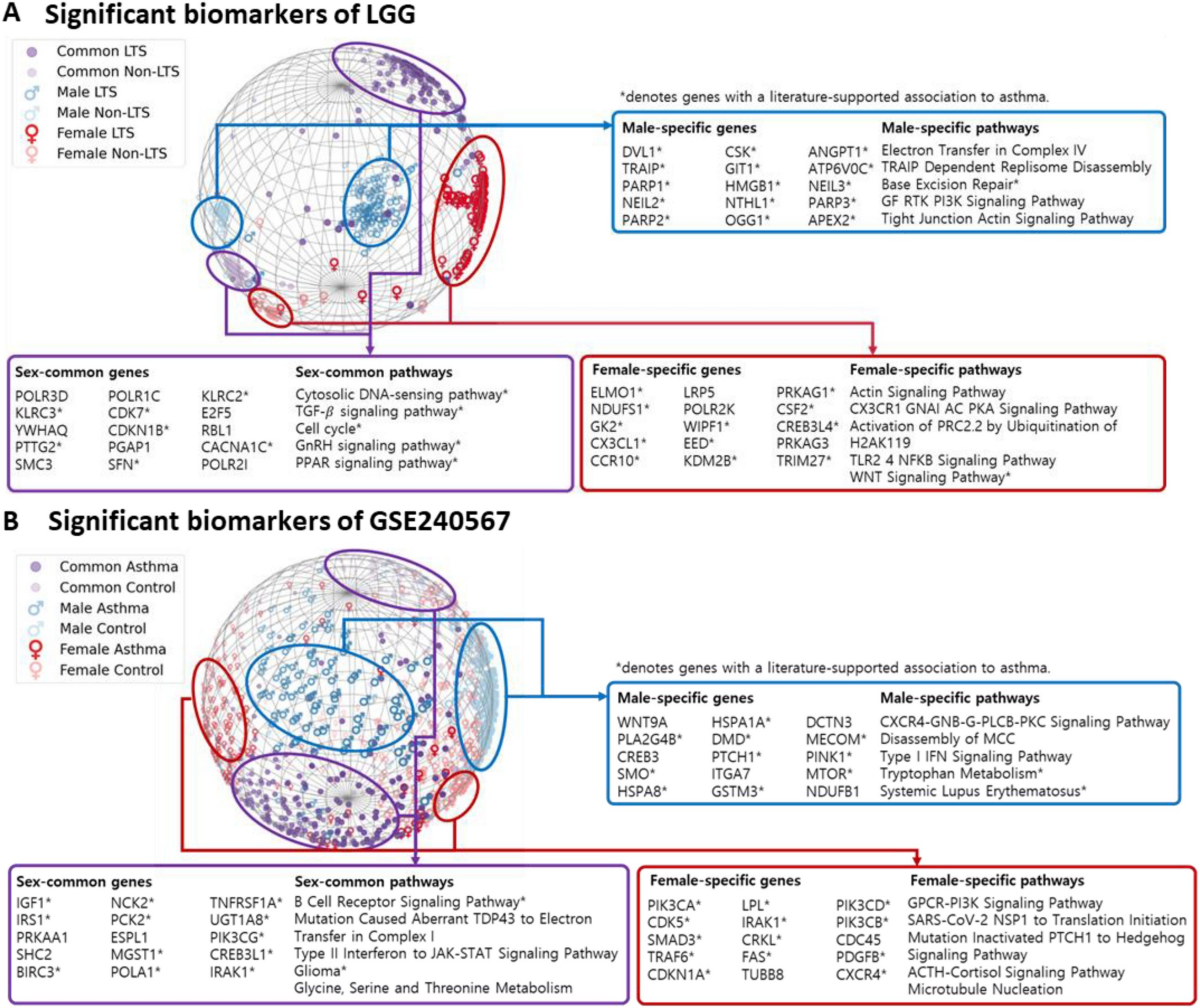
Visualization of FairHICON’s latent representations and identification of sex-specific and sex-common biomarkers. The figure displays the 3D-hypersphere latent spaces learned by FairHICON, illustrating the effective disentanglement of patient samples into distinct semantic subspaces: sex-common, male-specific, and female-specific. The connected tables display the top predictive genes and biological pathways. Features were selected based on having the highest statistical confidence (smallest adjusted *P*-values) and are sorted sequentially by their mean attribution importance score. (A) Analysis of the brain lower grade glioma (LGG) dataset. The spherical embedding demonstrates robust clustering of patients based on survival status (LTS versus non-LTS), separated within each sex-specific or common subspace. The connected tables list the most significant genes and biological pathways identified by the model, distinguishing unique male and female risk factors from shared predictive features. (B) Analysis of the asthma dataset (GSE240567). The visualization shows the separation between asthma and control samples across the three sex-defined subgroups. In both panels, the tables highlight the distinct genomic mechanisms driving predictions for each group. Genes and pathways marked with an asterisk (*) denote biomarkers with literature-supported associations to the respective disease, validating the biological relevance of the features discovered by FairHICON.

To validate the effectiveness of the representation, we visualized the latent space learned from the LGG and asthma (GSE240567) datasets (Fig. 2). The visualization shows that the model effectively separates the patient data into clear, biologically meaningful clusters that align with the outputs of the sex-common, male-specific, and female-specific encoders. Note that this visual separation primarily confirms that the hierarchical contrastive optimization achieved its geometric objective. While this demonstrates successful algorithmic disentanglement, independent biological validation of these partitions relies on subsequent prognostic evaluations. This clear separation provides strong empirical evidence that the hierarchical objective, combined with dedicated encoders, effectively partitioned the learned representations according to the underlying mechanisms of sex-related diseases.

To quantitatively verify that sex-related signals were fully excluded from the sex-common branch, we conducted an adversarial probing test across all datasets. We hypothesized that if the sex-common encoder successfully extracts sex-invariant features, an adversarial classifier trained solely on these representations would fail to predict the patient’s biological sex, yielding an Area Under the ROC Curve (AUROC) approximating 0.50 (random chance). Following the hierarchical contrastive optimization (across 10 experimental runs), we extracted the frozen latent representations from the sex-common encoder and trained a logistic regression classifier (via 5-fold cross-validation) to predict the patient’s sex. The adversarial probe yielded mean AUROC scores approximating random chance across all evaluated cohorts: 0.574±0.032 for LGG, 0.611±0.076 for LIHC, 0.527±0.022 for LUAD, and 0.606±0.029 for the GSE240567 asthma dataset. Contrasted with the high predictive accuracy for the primary clinical outcomes, this near-random adversarial performance confirms that the Level 2 hierarchical objective successfully restricts sex-specific signals from the shared representational space.

Finally, to elucidate the biological predictors driving these structured latent spaces, we performed feature attribution analysis on the representations derived from the three distinct encoders. We leveraged the biologically informed architecture to identify the most influential genes and pathways, further validating these findings through statistical analysis in the subsequent sections.

### 3.3 Identification of potential significant biomarkers

FairHICON uncovered distinct groups of biomarkers that were shared between sexes and those that were sex-specific in both the LGG and GSE240567 datasets, respectively, highlighting unique genomic mechanisms underlying disease in each group. To prioritize features for visualization (Fig. 2), statistically significant genes and pathways (FDR <0.01) were ranked by their mean absolute importance score. The comprehensive lists of all significant predictive features identified across the sex-common and sex-specific encoders, including their respective attribution scores and adjusted *P*-values, are provided in Tables 2–5, available as supplementary data at *Bioinformatics* online. Furthermore, we verified that our findings align with known biomarkers associated with brain glioma and asthma previously reported in the biological literature.

For the LGG analysis (Fig. 2A), the top sex-common features were driven by established mechanisms of cell cycle dysregulation, apoptosis, and extracellular matrix remodeling, highlighted by key genes like *CDK7*, *CDKN1B*, and *MMP2*, alongside the *Cell cycle* and *TGF-*β signaling pathways. In contrast, the male-specific encoder prioritized pathways related to DNA repair and cellular energetics (e.g. *Base Excision Repair*), driven by genes such as *TRAIP*, *PARP1*, and *NEIL2*, highlighting a potential male-specific reliance on genomic stability. Female-specific features were linked to cytoskeletal organization and immune signaling (e.g. *Actin*, *WNT*, and *CX3CR1-GNAI-AC-PKA* signaling), with influential genes including *ELMO1*, *CX3CL1*, and *NDUFS1*, suggesting distinct structural and inflammatory dependencies.

Similarly, for the asthma dataset (Fig. 2B), the sex-common encoder highlighted conserved metabolic and core immune processes (*B Cell Receptor* and *JAK-STAT* signaling), with prominent genes including *IGF1*, *IRS1*, and *TNFRSF1A*. Unique to males were immune-metabolic regulatory pathways (*Type I IFN signaling*, *Tryptophan Metabolism*) and genetic drivers like *WNT9A* and *PLA2G4B*. The female-specific encoder emphasized pathways related to hormonal and growth factor signaling. The prominence of the *ACTH-Cortisol pathway* and genes like *PIK3CA* and *SMAD3* perfectly aligns with known hormonal influences on asthma prevalence and severity in women.

### 3.4 Clinical validation of sex-specific genetic risk factors

**Figure 3:**
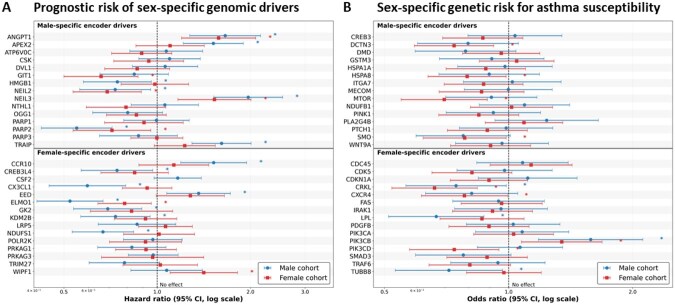
Quantitative assessment of sex-differential predictive features identified by FairHICON. (A) Prognostic risk analysis for LGG. Forest plots display the hazard ratios (HR) with 95% confidence intervals (CI) for top genes identified by the Male-Specific Encoder (top panel) and Female-Specific Encoder (bottom panel). Risks are stratified by sex for the male and female cohorts. HR >1 indicates increased risk of mortality, while HR <1 indicates a protective effect. (B) Genetic risk analysis for asthma susceptibility (GSE240567). Forest plots display the Odds Ratios (OR) with 95% CI for top genes, stratified by sex. OR >1 indicates increased susceptibility to asthma. The vertical dashed line represents the no effect threshold (HR/OR =1). An asterisk (*) marks results that remain statistically significant after Benjamini-Hochberg multiple testing correction (FDR-adjusted *P* < .05).

To validate the clinical relevance of the sex-differential predictive features identified by FairHICON (Fig. 2), we evaluated their association with clinical outcomes using sex-stratified regression analyses (Fig. 3). For the LGG dataset, we assessed the prognostic utility of the prioritized genes via univariable Cox Proportional Hazards (CPH) regression (Fig. 3A); for the asthma dataset, we evaluated disease susceptibility using univariable logistic regression to determine Odds Ratios (OR) (Fig. 3B).

For the prognosis of LGG cancer, the analysis revealed a substantial dissociation in risk attribution between the sexes. First, genomic factors prioritized by the male-specific encoder revealed distinct risk profiles. *NEIL3* and *ANGPT1* emerged as robust risk factors, exhibiting Hazard Ratios (HR) >1 in both sexes, with *NEIL3* showing a particularly strong association in males (HR ≈ 2.0, *P*<.05). *TRAIP* demonstrated a distinct sex-specific effect, functioning as a significant risk factor for males (HR >1, *P*<.05) while showing no statistically significant association in females (95% CI crossing 1.0). Conversely, genes such as *PARP2*, *PARP3*, and *OGG1* displayed protective associations (HR <1) in the male cohort, indicating their higher expression correlates with better survival outcomes. Second, we found that the identified female-specific drivers exerted particularly strong effects in women. *WIPF1* was a standout female-specific risk factor, exhibiting a significantly elevated HR in the female cohort compared to a negligible effect in males (HR >1, *P*<.05). *ELMO1* and *NDUFS1* were identified as robust protective factors (HR <1) for both sexes, with non-overlapping confidence intervals suggesting a consistent beneficial prognostic value.

Extending this validation to disease susceptibility in asthma (GSE240567), FairHICON identified several genes associated with increased asthma risk in males, including *PLA2G4B* and *NDUFB1*, both showing OR >1. *GSTM3* and *DMD* exhibited protective effects (OR <1) within the male cohort, suggesting that their expression may be associated with reduced susceptibility. Genes identified through the female encoder were strongly linked to asthma susceptibility. *PIK3CB* displayed a remarkably high odds ratio in the female cohort, indicating that it is a critical susceptibility factor for women (OR >1, *P*<.05). In contrast, *PIK3CD* and *CXCR4* were associated with a reduced susceptibility (OR <1, *P*<.05) in the female cohort, highlighting their potential protective roles in the pathology of female asthma.

### 3.5 Validation of sexual dimorphism via prognostic stratification

**Figure 4:**
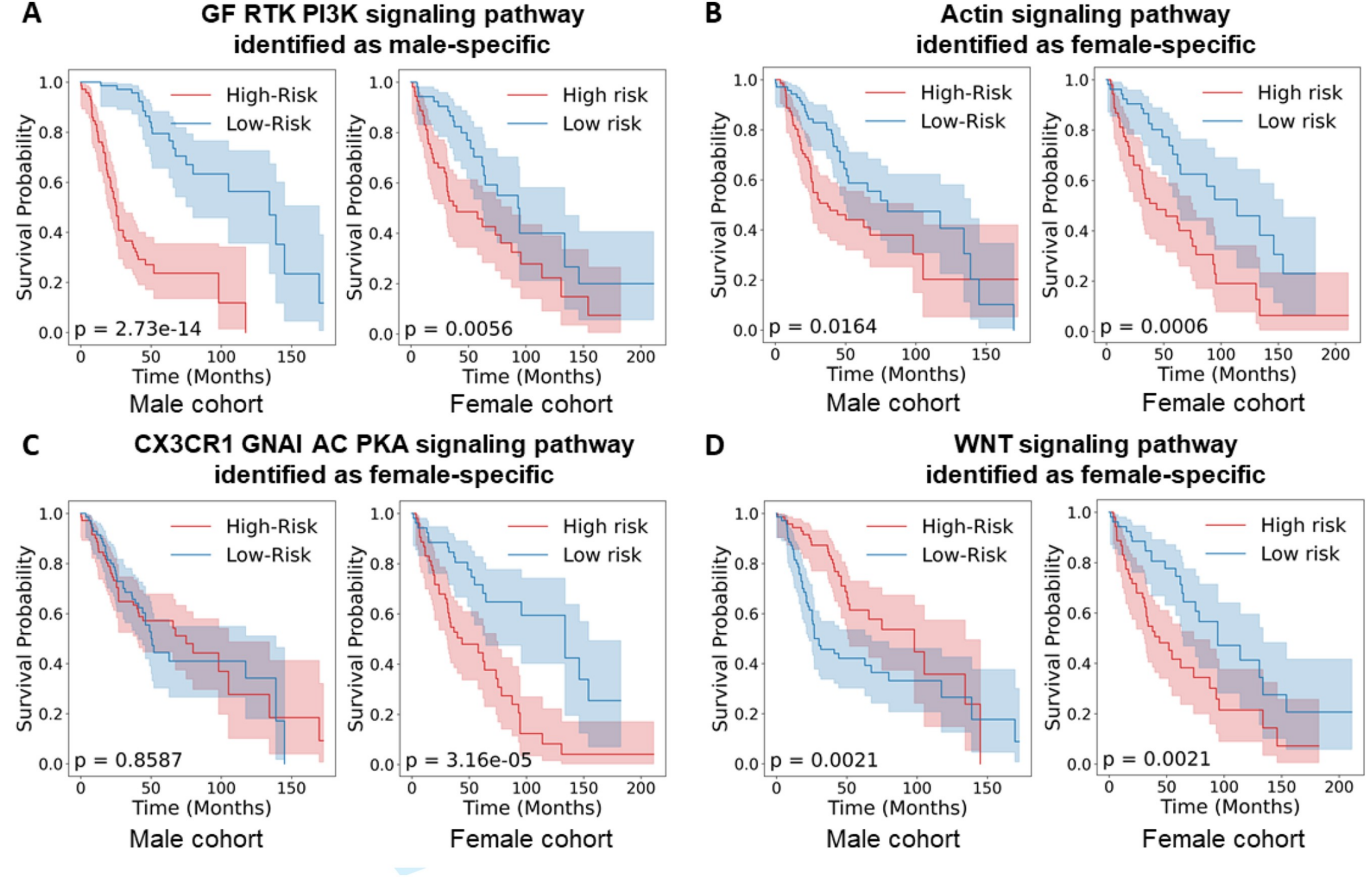
Validation of sexual dimorphism through comparative survival analysis. Patients were stratified into high-risk and low-risk groups based on the median activation of sex-specific pathways. The plots demonstrate that pathways stratify survival more effectively in the sex for which they were identified. (A) Male-specific driver: *GF RTK PI3K signaling pathway* exhibits a profound risk separation in the male cohort (*P* = 2.73×10^−14^), significantly outperforming the stratification observed in the female cohort (*P* = .0056). (B-D) Female-specific driver: *Actin signaling pathway* differentiates risk more significantly in females (*P* = .0006) than in males (*P* = .0164). *CX3CR1 GNAI AC PKA signaling pathway* functions as a female-exclusive prognostic marker (*P*<10^−14^), showing no stratification capability in the male cohort (*P* = .8587). *WNT signaling pathway* similarly displays superior prognostic stratification in the female cohort relative to the male cohort. *P*-values were calculated using the log-rank test.

To evaluate the predictive divergence identified by FairHICON, we assessed the prognostic distinctiveness of the prioritized pathways using Kaplan-Meier (KM) survival analysis (Fig. 4). The pathway activity score for a given patient was defined as the scalar activation value of the corresponding pathway node within the PASNet hidden layer. Patients were stratified into high-risk and low-risk groups using the median pathway activity score of their respective cohort as the cutoff threshold.

The *GF RTK PI3K signaling pathway* was identified as a significant risk factor by the male-specific encoder. Consistent with this attribution, the pathway exhibited exceptional prognostic power within the male cohort, yielding a clear separation between high-risk and low-risk groups (*P* = 2.73×10^−14^). In contrast, while the pathway remained statistically associated with survival in females (*P* = .0056), the stratification was orders of magnitude less distinct than in males. This disparity indicates that while this signaling axis is relevant to glioma generally, its specific dysregulation constitutes a disproportionately critical survival determinant for male physiology. This computational finding aligns with established experimental evidence indicating that male glioblastoma is disproportionately driven by cell cycle and growth factor signaling. Specifically, male glioblastoma cells exhibit significantly higher activity in the PI3K/mTOR pathway compared to female cells (Sponagel *et al.* 2022). Furthermore, glycolytic signatures downstream of this pathway yield significant prognostic value in males while failing to stratify survival in females (Ippolito *et al.* 2017), providing robust external validation for the male-specific risk attribution identified by FairHICON.

In contrast, pathways prioritized by the female-specific encoder, such as *Actin signaling*, *CX3CR1 GNAI AC PKA signaling*, and *WNT signaling*, demonstrated superior prognostic utility in women. The most definitive evidence of dimorphism was observed in the *CX3CR1 GNAI AC PKA signaling pathway*. This mechanism successfully stratified the female cohort with high significance (*P*<10^−4^) but failed entirely to predict survival outcomes in the male cohort (*P* = .8587). Similarly, the *Actin signaling pathway* achieved a highly significant separation in females (*P* = .0006) compared to a weaker association in males. This mirrors the findings that the survival of female glioblastoma is more closely related to invasive and structural signaling profiles (such as integrin and actin dynamics) than the predominant cell-cycle drivers in males (Yang *et al.* 2019).

These comparative survival analyzes confirm that the features extracted by FairHICON are not generic cancer markers, but rather distinct biological predictors that reflect the underlying sexual dimorphism of the disease. By correctly assigning the PI3K axis to males and structural/invasive pathways to females, the model demonstrates true predictive relevance, ensuring that clinical risk stratification is tailored to the distinct biological reality of each sex.

## 4 Discussion

While FairHICON successfully isolates sex-differential predictive features, our prognostic evaluation relies on independent sex-stratified analyses. Preliminary gene-by-sex interaction testing using unified models yielded nominal significance for several prioritized features; however, the results did not remain significant after false discovery rate correction due to the limited sample sizes inherent in HDLSS genomic cohorts. Consequently, the identified features serve as highly predictive, computationally derived hypotheses requiring formal causal validation in larger, multi-institutional datasets. A comprehensive discussion regarding these sample size constraints, the model’s theoretical robustness against external cohort shifts, and future extensions to single-cell and multi-omics data is provided in Supplementary Note 2, available as supplementary data at *Bioinformatics* online.

## Author contributions

Euiseong Ko (Conceptualization, Data curation, Methodology, Validation, Visualization, Writing), Sai Phani Parsa (Conceptualization, Validation), Sai Chandra Kosaraju (Conceptualization), Tesfaye B. Mersha (Supervision, Writing - review & editing), and Mingon Kang (Supervision, Writing - review & editing)

## Supplementary Material

btag237_Supplementary_Data

## Data Availability

The source code and data underlying this article are available in GitHub at https://github.com/datax-lab/FairHICON.

## References

[btag237-B1] Bourquard T , Lee K, Al-Ramahi I et al Functional variants identify sex-specific genes and pathways in Alzheimer’s disease. Nat Commun 2023;14:2765. 37179358 10.1038/s41467-023-38374-z PMC10183026

[btag237-B2] Chen T, Kornblith S, Norouzi M , et al A simple framework for contrastive learning of visual representations. In: *International Conference on Machine Learning*. Vienna, Austria (Virtual): PMLR, 2020, 1597–607.

[btag237-B3] Cook MB , McGlynn KA, Devesa SS et al Sex disparities in cancer mortality and survival. Cancer Epidemiol Biomarkers Prev 2011;20:1629–37. 21750167 10.1158/1055-9965.EPI-11-0246 PMC3153584

[btag237-B4] Gautam Y , Afanador Y, Abebe T et al Genome-wide analysis revealed sex-specific gene expression in asthmatics. Hum Mol Genet 2019;28:2600–14. 31095684 10.1093/hmg/ddz074 PMC6644163

[btag237-B5] Hao J , Kim Y, Kim T-K et al Pasnet: pathway-associated sparse deep neural network for prognosis prediction from high-throughput data. BMC Bioinformatics 2018;19:510–3. 30558539 10.1186/s12859-018-2500-z PMC6296065

[btag237-B6] Hartman RJG , Owsiany K, Ma L et al Sex-stratified gene regulatory networks reveal female key driver genes of atherosclerosis involved in smooth muscle cell phenotype switching. Circulation 2021;143:713–26. 33499648 10.1161/CIRCULATIONAHA.120.051231 PMC7930467

[btag237-B7] Hases L , Ibrahim A, Chen X et al The importance of sex in the discovery of colorectal cancer prognostic biomarkers. Int J Mol Sci 2021;22:1354. 33572952 10.3390/ijms22031354 PMC7866425

[btag237-B8] Ippolito JE , Yim AK-Y, Luo J et al Sexual dimorphism in glioma glycolysis underlies sex differences in survival. JCI Insight 2017;2:e92142. 28768910 10.1172/jci.insight.92142 PMC5543918

[btag237-B9] Jun T , Nirenberg S, Weinberger T et al Analysis of sex-specific risk factors and clinical outcomes in covid-19. Commun Med (Lond) 2021;1:3. 35602223 10.1038/s43856-021-00006-2 PMC9053255

[btag237-B10] Kim H-I , Lim H, Moon A. Sex differences in cancer: epidemiology, genetics and therapy. Biomol Ther (Seoul) 2018;26:335–42. 29949843 10.4062/biomolther.2018.103 PMC6029678

[btag237-B11] Ko E , Kim Y, Shokoohi F et al Spin: sex-specific and pathway-based interpretable neural network for sexual dimorphism analysis. Brief Bioinform 2024;25:bbae239. 38807262 10.1093/bib/bbae239 PMC11133003

[btag237-B12] Lopes-Ramos CM , Kuijjer ML, Ogino S et al Gene regulatory network analysis identifies sex-linked differences in colon cancer drug metabolism. Cancer Res 2018;78:5538–47. 30275053 10.1158/0008-5472.CAN-18-0454 PMC6169995

[btag237-B13] Lopes-Ramos CM , Chen C-Y, Kuijjer ML et al Sex differences in gene expression and regulatory networks across 29 human tissues. Cell Rep 2020;31:107795. 32579922 10.1016/j.celrep.2020.107795 PMC7898458

[btag237-B14] Ober C , Loisel DA, Gilad Y et al Sex-specific genetic architecture of human disease. Nat Rev Genet 2008;9:911–22. 19002143 10.1038/nrg2415 PMC2694620

[btag237-B15] Oliva M , Muñoz-Aguirre M, Kim-Hellmuth S, GTEx Consortium et al The impact of sex on gene expression across human tissues. Science (1979) 2020;369:eaba3066. 10.1126/science.aba3066 PMC8136152 32913072

[btag237-B16] Park S, Lee J, Lee P et al Fair contrastive learning for facial attribute classification. In: *Proceedings of the IEEE/CVF Conference on Computer Vision and Pattern Recognition.* New Orleans, LA, USA: IEEE, 2022, 10389–98.

[btag237-B17] Postma DS. Gender differences in asthma development and progression. Gender Med, 2007;4:S133–46. 10.1016/s1550-8579(07)80054-4 18156099

[btag237-B18] Rubin JB , Lagas JS, Broestl L et al Sex differences in cancer mechanisms. Biol Sex Differ 2020;11:17. 32295632 10.1186/s13293-020-00291-x PMC7161126

[btag237-B19] Siegel RL , Miller KD, Fuchs HE et al Cancer statistics, 2021. CA Cancer J Clin 2021;71:7–33. 33433946 10.3322/caac.21654

[btag237-B20] Sponagel J , Johnston H, Gass H et al Csig-19. the pi3k/akt/mtor signaling Cascade may contribute to sex differences in glioblastoma. Neuro-Oncology 2022;24:vii43.

[btag237-B21] Wang X , Chen Y, Zhu W. A survey on curriculum learning. IEEE Trans Pattern Anal Mach Intell 2021;44:4555–76. 10.1109/TPAMI.2021.3069908 33788677

[btag237-B22] Yang W , Warrington NM, Taylor SJ et al Sex differences in GBM revealed by analysis of patient imaging, transcriptome, and survival data. Sci Transl Med 2019;11:eaao5253. 30602536 10.1126/scitranslmed.aao5253 PMC6502224

[btag237-B23] Zheng D , Trynda J, Williams C et al Sexual dimorphism in the incidence of human cancers. BMC Cancer 2019;19:684. 31299933 10.1186/s12885-019-5902-z PMC6625025

