# Supplementary material for: Fairness-aware supervised hierarchical contrastive semantic learning for sexual dimorphism analysis
Source: Bioinformatics. 2026 Jul 7;42(Suppl 1):btag237. doi: 10.1093/bioinformatics/btag237 (PMC13340225; doi:10.1093/bioinformatics/btag237)
Supplement: btag237_Supplementary_Data [file btag237_supplementary_data.pdf]

Fairness-aware Supervised Hierarchical Contrastive Semantic Learning for Sexual Dimorphism Analysis

Euiseong Ko[1], Sai Phani Parsa[2], Sai Chandra Kosaraju[3], Tesfaye B. Mersha[4*], and Mingon Kang[2*]

[1]Department of Biomedical Informatics and Data Science, Heersink School of Medicine, University of Alabama at Birmingham, AL, USA,

[2]Department of Computer Science, University of Nevada, Las Vegas, NV, USA

[3]Department of Computer Science, California State Polytechnic University, CA, USA

[4]Department of Medicine, Indiana University School of Medicine, IN, USA

* Corresponding authors. tmersha@iu.edu, mingon.kang@unlv.edu

Supplementary Note S1: Theoretical Mapping of SHCSL to Fairness Metrics

Within FairHICON, fairness is formally defined as the strict geometric disentanglement of structural mechanisms. The Level 1 hierarchy maximizes global predictive accuracy by clustering samples into two primary groups based strictly on clinical outcome (disease vs. control). Because this level is demographic-agnostic, standard models remain susceptible to algorithmic bias. FairHICON resolves this through the Level 2 hierarchy, which serves as the mathematical mechanism for fairness by disentangling these primary clusters into six distinct semantic subgroups (sex-common, male-specific, and female-specific for each clinical outcome). By actively applying adaptive weighting to penalize intra-class overlap among these six groups, the model is effectively prevented from collapsing predictions onto a spurious, sex-biased proxy. This explicit structural disentanglement ensures the downstream classifier evaluates distinct features for each sex. Consequently, this geometry naturally symmetricizes the error margins across groups (minimizing Equalized Odds Difference) and decorrelates the overall prediction rate from the sensitive attribute by forcing the model to rely on true biological dimorphism (minimizing Demographic Parity Difference).

Importantly, achieving statistical parity in predictions (DPD and EOD) does not fully guarantee fairness in the context of sexual dimorphism. A comprehensively fair translational model should also achieve representational fairness by accurately elucidating the distinct pathogenic drivers for each sex. While our quantitative metrics demonstrate that FairHICON resolves algorithmic imbalance, the evaluation of latent space disentanglement and clinical validation (Sections 3.2--3.5) serves as our post-hoc evaluation of biological fairness, demonstrating that the model successfully identifies clinically relevant sex-specific biomarkers rather than merely equalizing predictive performance.

Supplementary Note S2: Extended Discussion on Model Generalizability and Limitations

Interaction Testing: Comparing stratified risk ratios does not formally establish sex-differential effects. In our preliminary gene-by-sex interaction testing using unified survival and classification models, several prioritized features (e.g., *CX3CL1* and *ELMO1* in LGG; *PIK3CD* in asthma) exhibited nominal statistical significance for the interaction term. However, they did not retain significance following false discovery rate correction. This highlights a common limitation in genomic studies: the available sample sizes often lack the statistical sensitivity required to detect significant interaction effects after stringent multiple-testing corrections. Consequently, the identified genes and pathways should be interpreted as highly predictive, computationally derived hypotheses that require formal causal validation through covariate-adjusted interaction testing in larger datasets that are not constrained by HDLSS limitations.

Cohort and Demographic Shifts: While our evaluation demonstrates internal stability across repeated random splits, performance and fairness metrics can degrade when a model is applied to new populations due to varying genetic backgrounds, differing clinical environments, or technical batch effects. Theoretically, FairHICON's hierarchical contrastive objective provides inherent robustness against certain domain shifts by isolating fundamental biological predictors rather than relying on superficial, cohort-specific noise. Regarding changes in demographic composition, the group-wise normalization and adaptive weighting mechanisms within the SHCSL objective are mathematically designed to maintain stable gradient contributions regardless of the underlying sex ratio. However, extreme demographic skew in an external deployment could still challenge the model's ability to symmetrically map the minority manifold, making validation on independent, multi-institutional datasets an important next step for clinical translation.

Methodological Limitations and Future Directions: The analysis of bulk gene expression data inherently involves the High-Dimensional Low-Sample Size (HDLSS) challenge, which can affect the robustness and generalizability of the findings. Although we employed preprocessing strategies and pathway integration to mitigate overfitting, this risk remains an inherent challenge in genomic studies. Furthermore, reliance on predefined pathway databases can limit the discovery of novel biological connections outside existing knowledge graphs. Future work will aim to address these constraints by applying FairHICON to single-cell RNA-Seq data, providing higher-resolution insights into cellular heterogeneity. Extending the framework to integrate multi-omics data (including epigenomics and proteomics) will provide a more comprehensive understanding of the molecular landscape of sexual dimorphism.

Supplementary Table S1. Predictive performance of the Naive Sex-Stratified PASNet Baseline across four datasets. Models were trained independently on male-only and female-only cohorts utilizing the same class-weighting scheme as the primary models. Overall performance was calculated by recombining the predictions from the independent models to simulate the performance of a fully fragmented dataset.

Dataset	Metric	Male-Only Model	Female-Only Model	Overall (Combined)
LGG	AUROC	0.922 ± 0.044	0.731 ± 0.137	0.845 ± 0.069
	AUPRC	0.906 ± 0.066	0.759 ± 0.132	0.821 ± 0.097
LIHC	AUROC	0.697 ± 0.083	0.521 ± 0.138	0.644 ± 0.064
	AUPRC	0.714 ± 0.094	0.527 ± 0.121	0.654 ± 0.084
LUAD	AUROC	0.655 ± 0.103	0.542 ± 0.105	0.565 ± 0.058
	AUPRC	0.676 ± 0.130	0.586 ± 0.107	0.602 ± 0.060
GSE240567	AUROC	0.692 ± 0.073	0.721 ± 0.038	0.698 ± 0.038
	AUPRC	0.696 ± 0.083	0.756 ± 0.042	0.723 ± 0.041

Supplementary Table S2. Top 30 Predictive Genes by Subgroup (LGG Cohort)

Common Gene	t-stat	p-val(FDR)	Importance	Male Gene	t-stat	p-val(FDR)	Importance	Female Gene	t-stat	p-val(FDR)	Importance
CDK7	1259.08	< 1.00E-300 (< 1.00E-300)	1.11E-06	NEIL3	858.04	2.29E-262 (4.39E-260)	1.20E-06	CREB3L4	667.64	1.05E-190 (5.57E-188)	2.14E-06
YWHAQ	1235.19	< 1.00E-300 (< 1.00E-300)	9.91E-07	DVL1	951.14	1.25E-268 (6.00E-265)	1.14E-06	PRKAG3	645.5	3.49E-189 (1.39E-186)	1.84E-06
POLR2I	1067.81	< 1.00E-300 (< 1.00E-300)	9.45E-07	FGF23	795.8	8.66E-258 (1.38E-255)	9.37E-07	WIPF1	669.17	8.26E-191 (4.94E-188)	1.21E-06

CDKN1B	1224.8	< 1.00E-300 (< 1.00E-300)	9.43E-07	DNAH11	797.98	5.91E-258 (9.77E-256)	7.91E-07	ITGA10	592.51	2.58E-185 (4.41E-183)	1.15E-06
POLR2K	1060.92	< 1.00E-300 (< 1.00E-300)	9.37E-07	NEIL1	858.04	2.29E-262 (4.39E-260)	7.39E-07	LRP5	729.92	9.85E-195 (2.36E-191)	1.05E-06
CACNA1C	1122.08	< 1.00E-300 (< 1.00E-300)	9.06E-07	GIT1	890.33	1.30E-264 (1.25E-261)	6.62E-07	POLR1A	591.77	2.94E-185 (4.85E-183)	9.75E-07
POLR1C	1537.61	< 1.00E-300 (< 1.00E-300)	7.66E-07	MBD4	858.04	2.29E-262 (4.39E-260)	6.32E-07	NDUFS1	721.17	3.45E-194 (5.03E-191)	9.38E-07
MMP2	1064.91	< 1.00E-300 (< 1.00E-300)	7.58E-07	HMGB1P1	858.04	2.29E-262 (4.39E-260)	6.24E-07	AKR1C4	607.2	2.02E-186 (4.03E-184)	9.28E-07
IL33	1035.72	< 1.00E-300 (< 1.00E-300)	7.39E-07	HMGB1	858.04	2.29E-262 (4.39E-260)	6.20E-07	CDK5	593.99	1.99E-185 (3.53E-183)	9.04E-07
PTTG2	1140.25	< 1.00E-300 (< 1.00E-300)	6.66E-07	CSK	918.28	1.72E-266 (4.12E-263)	5.97E-07	EED	648.95	2.01E-189 (8.74E-187)	8.92E-07
HBEGF	1039.78	< 1.00E-300 (< 1.00E-300)	6.62E-07	MPG	858.04	2.29E-262 (4.39E-260)	5.79E-07	IRS2	601.86	5.06E-186 (9.70E-184)	8.50E-07
MMP1	1049.52	< 1.00E-300 (< 1.00E-300)	5.97E-07	ANGPT1	896.51	4.95E-265 (7.90E-262)	5.36E-07	RAB5A	612.79	7.79E-187 (1.70E-184)	6.31E-07
POLR3D	1544.77	< 1.00E-300 (< 1.00E-300)	5.93E-07	ATP6V0C	878.66	8.25E-264 (6.58E-261)	5.28E-07	BCORL1	631.17	3.61E-188 (9.09E-186)	4.90E-07

CHRD	1029.69	< 1.00E-300 (< 1.00E-300)	5.63E-07	APEX2	858.04	2.29E-262 (4.39E-260)	4.81E-07	RFC5	591.26	3.21E-185 (5.12E-183)	4.42E-07
KLRC3	1301.76	< 1.00E-300 (< 1.00E-300)	5.27E-07	OGG1	858.04	2.29E-262 (4.39E-260)	3.86E-07	CSF2	705.35	3.46E-193 (2.96E-190)	4.33E-07
E2F5	1242.89	< 1.00E-300 (< 1.00E-300)	4.39E-07	APEX1	858.04	2.29E-262 (4.39E-260)	3.59E-07	CX3CL1	654.28	8.58E-190 (4.11E-187)	4.10E-07
ZBP1	1035.72	< 1.00E-300 (< 1.00E-300)	4.23E-07	ATP6V1E1	799.79	4.30E-258 (7.36E-256)	3.20E-07	TRIM27	631.17	3.61E-188 (9.09E-186)	3.99E-07
LTBP1	1029.69	< 1.00E-300 (< 1.00E-300)	4.23E-07	XRCC1	859.83	1.71E-262 (4.39E-260)	2.84E-07	POLR2K	704.88	3.71E-193 (2.96E-190)	3.70E-07
TREX1	1035.72	< 1.00E-300 (< 1.00E-300)	4.03E-07	PARP1	858.04	2.29E-262 (4.39E-260)	2.66E-07	GK2	683.74	8.80E-192 (6.02E-189)	3.63E-07
SFN	1105.2	< 1.00E-300 (< 1.00E-300)	3.82E-07	CETN2	822.22	8.96E-260 (1.59E-257)	2.61E-07	ANGPT1	620.01	2.30E-187 (5.52E-185)	2.95E-07
RBL1	1211.49	< 1.00E-300 (< 1.00E-300)	2.60E-07	NTHL1	858.04	2.29E-262 (4.39E-260)	2.27E-07	AREG	616.86	3.91E-187 (8.93E-185)	2.84E-07
HSPA5	1063.31	< 1.00E-300 (< 1.00E-300)	2.45E-07	TDG	858.04	2.29E-262 (4.39E-260)	2.18E-07	PRKAG1	719.8	4.20E-194 (5.03E-191)	2.82E-07
PGAP1	1133.81	< 1.00E-300 (< 1.00E-300)	2.41E-07	TRAIP	892.05	9.94E-265 (1.19E-261)	2.09E-07	ELMO1	854.69	7.36E-202 (3.52E-198)	2.61E-07

AIM2	1035.72	< 1.00E-300 (< 1.00E-300)	2.24E-07	POLL	858.04	2.29E-262 (4.39E-260)	1.72E-07	CCL23	601.45	5.44E-186 (1.00E-183)	2.48E-07
PIGX	1038.18	< 1.00E-300 (< 1.00E-300)	2.07E-07	NEIL2	858.04	2.29E-262 (4.39E-260)	1.53E-07	CCR10	633.52	2.45E-188 (9.03E-186)	2.31E-07
ID1	1029.69	< 1.00E-300 (< 1.00E-300)	1.97E-07	SMUG1	858.04	2.29E-262 (4.39E-260)	1.39E-07	BCOR	631.17	3.61E-188 (9.09E-186)	2.29E-07
NOG	1029.69	< 1.00E-300 (< 1.00E-300)	1.87E-07	CLDN25	825.46	5.17E-260 (9.52E-258)	8.61E-08	CXCL13	612.06	8.81E-187 (1.83E-184)	2.11E-07
SMC3	1106.47	< 1.00E-300 (< 1.00E-300)	1.74E-07	PARP3	858.04	2.29E-262 (4.39E-260)	5.60E-08	PCGF1	631.17	3.61E-188 (9.09E-186)	1.81E-07
KLRC2	1317.51	< 1.00E-300 (< 1.00E-300)	1.62E-07	PARP2	858.04	2.29E-262 (4.39E-260)	4.10E-09	USP7	631.17	3.61E-188 (9.09E-186)	1.11E-07
PIGA	1065.89	< 1.00E-300 (< 1.00E-300)	1.57E-07	MUTYH	858.04	2.29E-262 (4.39E-260)	3.41E-09	KDM2B	631.17	3.61E-188 (9.09E-186)	1.07E-08

Supplementary Table S3. Top 30 Predictive Pathways by Subgroup (LGG Cohort)

Common Pathway	t-stat	p-val(FDR)	Importance	Male Pathway	t-stat	p-val(FDR)	Importance	Female Pathway	t-stat	p-val(FDR)	Importance
Nitrogen metabolism	691.3	< 1.00E-300 (< 1.00E-300)	3.41E-04	Epithelial cell signaling in helicobacter pylori infection	580.18	1.40E-238 (2.91E-237)	3.46E-04	FZD7 overexpression to WNT signaling pathway	547.2	1.01E-181 (7.96E-180)	3.93E-04

Organization of the inner kinetochore	702.81	< 1.00E-300 (< 1.00E-300)	3.19E-04	Disassembly of MCC	603.41	5.75E-241 (1.51E-239)	2.62E-04	HCMV US28 to GNAI AC PKA Signaling Pathway	436.44	1.63E-171 (2.81E-170)	3.27E-04
AGE-RAGE signaling pathway	699.46	< 1.00E-300 (< 1.00E-300)	3.10E-04	Base excision repair	879.52	7.19E-264 (9.47E-262)	2.60E-04	HIV TAT to TLR2 4 NFKB Signaling Pathway	620.96	1.97E-187 (1.94E-185)	3.12E-04
Cell cycle	1011.77	< 1.00E-300 (< 1.00E-300)	2.78E-04	Organization of the outer kinetochore	712.12	4.91E-251 (1.94E-249)	2.48E-04	HCMV US28 to GNAQ PLCB G CALCINEURIN Signaling Pathway	476.07	1.95E-175 (4.05E-174)	2.98E-04
Lysine degradation	760.02	< 1.00E-300 (< 1.00E-300)	2.75E-04	Mutation inactivated PTCH1 to hedgehog signaling pathway	692.25	2.58E-249 (9.25E-248)	2.47E-04	Escherichia eae tir to actin signaling pathway	760.48	1.38E-196 (5.46E-194)	2.77E-04
Cytosolic DNA sensing pathway	1055.86	< 1.00E-300 (< 1.00E-300)	2.56E-04	GF RTK PI3K signaling pathway	851.42	6.78E-262 (6.70E-260)	2.38E-04	Glycerolipid metabolism	452.66	3.69E-173 (6.93E-172)	2.71E-04

RTK PLCG ITPR signaling pathway	654.8	< 1.00E-300 (< 1.00E-300)	2.24E-04	TRAIP dependent replisome disassembly	894.89	6.37E-265 (1.26E-262)	2.28E-04	Activation of PRC2.2 by ubiquitination of H2AK119	631.72	3.29E-188 (4.34E-186)	2.67E-04
TGF-beta signaling pathway	1040.08	< 1.00E-300 (< 1.00E-300)	2.12E-04	Mutation caused aberrant HTT to anterograde axonal transport	539.01	4.16E-234 (6.33E-233)	2.25E-04	Asthma	541.54	2.97E-181 (1.95E-179)	2.65E-04
Glycosylphosphatidylinositol GPI-anchor biosynthesis	824.14	< 1.00E-300 (< 1.00E-300)	2.10E-04	HTLV 1 TAX to spindle assembly checkpoint signaling	773.44	4.68E-256 (2.64E-254)	2.09E-04	CX3CR1 GNAI AC PKA Signaling Pathway	632.61	2.85E-188 (4.34E-186)	2.46E-04
PPAR signaling pathway	918.2	< 1.00E-300 (< 1.00E-300)	2.10E-04	Vibrio cholerae infection	679.64	3.38E-248 (1.03E-246)	2.02E-04	B Cell receptor signaling pathway	502.28	7.42E-178 (2.27E-176)	2.33E-04
Assembly and trafficking of telomerase	761.92	< 1.00E-300 (< 1.00E-300)	2.01E-04	O glycan biosynthesis	543.51	1.30E-234 (2.23E-233)	1.94E-04	Tight Junction Actin Signaling Pathway	502.68	6.84E-178 (2.27E-176)	2.19E-04
GnRH signaling pathway	954.22	< 1.00E-300 (< 1.00E-300)	1.97E-04	BCR PLCG calcineurin signaling pathway	570.6	1.44E-237 (2.84E-236)	1.93E-04	ITGA B FAK CAS Signaling Pathway	502.93	6.48E-178 (2.27E-176)	2.18E-04

B Cell receptor signaling pathway	696.84	< 1.00E-300 (< 1.00E-300)	1.92E-04	Global genome NER	823.84	6.80E-260 (4.48E-258)	1.86E-04	KEAP1 NRF2 Signaling Pathway	514.97	5.54E-179 (2.74E-177)	1.98E-04
Global genome NER	776.8	< 1.00E-300 (< 1.00E-300)	1.91E-04	IFN RIPK1 3 signaling pathway	744.05	1.06E-253 (5.23E-252)	1.83E-04	Chemokine signaling pathway	487.21	1.76E-176 (4.10E-175)	1.97E-04
Cardiac type VGCC RYR signaling	880.37	< 1.00E-300 (< 1.00E-300)	1.87E-04	Tight Junction Actin Signaling Pathway	826.26	4.52E-260 (3.57E-258)	1.82E-04	PRNP PI3K NCAM1 FYN FAK Signaling Pathway	494.57	3.70E-177 (9.75E-176)	1.93E-04
LPAR GNB G rho signaling pathway	874.92	< 1.00E-300 (< 1.00E-300)	1.72E-04	TNF p38 signaling pathway	684.46	1.26E-248 (4.14E-247)	1.81E-04	Regulation of GF RTK RAS erk signaling ubiquitination of RTK by CBL	484.31	3.28E-176 (7.20E-175)	1.91E-04
Antigen processing and presentation	700.5	< 1.00E-300 (< 1.00E-300)	1.68E-04	Arrhythmogenic right ventricular cardiomyopathy ARVC	530.32	4.05E-233 (5.71E-232)	1.77E-04	Nicotinate and nicotinamide metabolism	502.24	7.48E-178 (2.27E-176)	1.84E-04
Endocytosis	682.08	< 1.00E-300 (< 1.00E-300)	1.67E-04	Melanogenesis	633.88	5.83E-244 (1.64E-242)	1.73E-04	TLR5 NFKB Signaling Pathway	491.46	7.14E-177 (1.76E-175)	1.80E-04

Hematopoietic cell lineage	686.5	< 1.00E-300 (< 1.00E-300)	1.60E-04	Mutation caused aberrant abeta to electron transfer in complex iv	920.65	1.20E-266 (4.74E-264)	1.69E-04	GF RTK RAS signaling pathway	470.43	6.73E-175 (1.33E-173)	1.80E-04
IL1 IL1R p38 signaling pathway	725.55	< 1.00E-300 (< 1.00E-300)	1.60E-04	Notch signaling pathway	545.88	7.07E-235 (1.27E-233)	1.58E-04	Mutation caused aberrant abeta to anterograde axonal transport	416.95	1.89E-169 (2.57E-168)	1.76E-04
Retrograde axonal transport	729.71	< 1.00E-300 (< 1.00E-300)	1.58E-04	Escherichia eae tir to actin signaling pathway	561.33	1.42E-236 (2.68E-235)	1.56E-04	Homologous recombination in ICLR	523.48	1.01E-179 (5.70E-178)	1.75E-04
PINK Parkin mediated autophagosome formation	827.4	< 1.00E-300 (< 1.00E-300)	1.54E-04	ITGA b rhogef rhoa signaling pathway	539.71	3.46E-234 (5.47E-233)	1.52E-04	RNA polymerase	403.23	6.11E-168 (8.04E-167)	1.67E-04
Homologous recombination in ICLR	696.92	< 1.00E-300 (< 1.00E-300)	1.53E-04	Pentose and glucuronate interconversions	588.29	2.01E-239 (4.95E-238)	1.51E-04	Organization of the outer kinetochore	508.09	2.25E-178 (9.85E-177)	1.62E-04
Natural killer cell mediated cytotoxicity	654.85	< 1.00E-300 (< 1.00E-300)	1.47E-04	CD80 CD86 CTLA4 PP2A signaling pathway	587.67	2.32E-239 (5.39E-238)	1.51E-04	Dorsoventral axis formation	429.23	9.24E-171 (1.40E-169)	1.61E-04

Epithelial cell signaling in helicobacter pylori infection	747.22	< 1.00E-300 (< 1.00E-300)	1.36E-04	Mutation caused aberrant TUBA4A to retrograde axonal transport	712.33	4.72E-251 (1.94E-249)	1.44E-04	JAK-STAT signaling pathway	431.9	4.85E-171 (7.98E-170)	1.50E-04
IL6 Family to JAK-STAT signaling pathway	684.68	< 1.00E-300 (< 1.00E-300)	1.32E-04	Mutation caused aberrant SOD1 to retrograde axonal transport	522.84	2.95E-232 (4.01E-231)	1.37E-04	Amyotrophic lateral sclerosis als	494.97	3.41E-177 (9.61E-176)	1.47E-04
HIV GP120 to CXCR4 GNAQ PLCB G CALCINEURIN	677	< 1.00E-300 (< 1.00E-300)	1.31E-04	Regulation of actin cytoskeleton	586.61	2.99E-239 (6.57E-238)	1.35E-04	DNA replication termination	430.37	7.02E-171 (1.11E-169)	1.45E-04
Regulation of actin cytoskeleton	660.51	< 1.00E-300 (< 1.00E-300)	1.22E-04	TSH TG signaling pathway	530.9	3.47E-233 (5.08E-232)	1.34E-04	Mutation inactivated RASD1 to CRHR PKA ACTH signaling pathway	449.78	7.15E-173 (1.28E-171)	1.36E-04
GF RTK RAS PI3K signaling pathway	758.38	< 1.00E-300 (< 1.00E-300)	1.20E-04	Steroid hormone biosynthesis	520.83	5.06E-232 (6.66E-231)	1.29E-04	BMP signaling pathway	421.46	6.17E-170 (8.71E-169)	1.36E-04

Leukocyte transendothelial migration	775.98	< 1.00E-300 (< 1.00E-300)	9.41E-05	Medicus env factor Zn to anterograde axonal transport	542.25	1.80E-234 (2.96E-233)	1.26E-04	PPAR signaling pathway	422.28	5.05E-170 (7.38E-169)	1.06E-04

Supplementary Table S4. Top 30 Predictive Genes by Subgroup (Asthma Cohort)

Common Gene	t-stat	p-val(FDR)	Importance	Male Gene	t-stat	p-val(FDR)	Importance	Female Gene	t-stat	p-val(FDR)	Importance
SOS1	11.94	2.64E-29 (6.61E-27)	1.07E-08	ALDH1B1	6.96	4.62E-11 (9.04E-09)	4.36E-09	PIK3CB	9.75	6.70E-20 (6.89E-17)	6.08E-09
PIK3CG	12.04	1.08E-29 (5.56E-27)	1.06E-08	WNT9A	7.63	8.50E-13 (3.82E-09)	3.72E-09	PIK3CA	9.66	1.38E-19 (6.89E-17)	3.74E-09
CREB3L1	11.99	1.64E-29 (6.16E-27)	6.35E-09	ITGA7	7.09	2.14E-11 (7.40E-09)	3.54E-09	PIK3CD	9.75	6.94E-20 (6.89E-17)	3.65E-09
MET	11.91	3.54E-29 (7.25E-27)	5.36E-09	ITGA10	6.96	4.61E-11 (9.04E-09)	3.40E-09	DVL1	9.34	1.50E-18 (2.33E-16)	3.29E-09
WNT10A	11.76	1.49E-28 (2.18E-26)	5.31E-09	PTCH1	7.15	1.51E-11 (7.40E-09)	3.26E-09	TUBB8	9.54	3.35E-19 (1.08E-16)	2.99E-09
PPP3CC	11.93	3.02E-29 (6.80E-27)	5.21E-09	CREB3	7.14	1.55E-11 (7.40E-09)	2.85E-09	SMAD3	9.67	1.24E-19 (6.89E-17)	2.78E-09
TRAF6	11.83	7.59E-29 (1.42E-26)	4.70E-09	ANAPC7	7.01	3.40E-11 (8.90E-09)	2.48E-09	TRAF6	9.61	1.92E-19 (8.65E-17)	2.68E-09

TNFRSF1A	12.19	2.42E-30 (2.72E-27)	4.44E-09	PFKM	6.97	4.27E-11 (9.04E-09)	2.30E-09	PDGFB	9.6	2.19E-19 (8.71E-17)	2.55E-09
UGT1A8	12.09	6.53E-30 (4.35E-27)	4.40E-09	PLA2G4B	7.3	6.05E-12 (7.14E-09)	2.25E-09	CDKN1A	9.57	2.64E-19 (9.14E-17)	2.53E-09
APAF1	11.79	1.09E-28 (1.81E-26)	4.35E-09	BDNF	6.93	5.26E-11 (9.46E-09)	2.13E-09	TUBB2B	9.46	5.92E-19 (1.57E-16)	2.47E-09
POLD1	11.96	2.16E-29 (6.40E-27)	4.13E-09	SMO	7.09	2.07E-11 (7.40E-09)	2.13E-09	CDK5	9.69	1.08E-19 (6.89E-17)	2.41E-09
SHC2	12.01	1.39E-29 (6.16E-27)	4.01E-09	PINK1	7.13	1.64E-11 (7.40E-09)	2.06E-09	IRAK1	9.73	7.81E-20 (6.89E-17)	2.28E-09
WNT7B	11.77	1.29E-28 (2.01E-26)	3.76E-09	MTOR	7.13	1.73E-11 (7.40E-09)	2.02E-09	CRKL	9.85	3.14E-20 (6.89E-17)	2.25E-09
IRS1	12.22	1.94E-30 (2.72E-27)	3.64E-09	RHOA	6.93	5.23E-11 (9.46E-09)	1.92E-09	NCK2	9.41	8.65E-19 (1.95E-16)	2.25E-09
IGF1	12.4	3.10E-31 (1.16E-27)	3.37E-09	WNT5A	6.96	4.61E-11 (9.04E-09)	1.91E-09	CXCR4	9.52	3.88E-19 (1.16E-16)	2.14E-09
IRAK1	11.97	2.06E-29 (6.40E-27)	3.35E-09	DCTN3	7.3	6.34E-12 (7.14E-09)	1.87E-09	DPM2	9.35	1.43E-18 (2.32E-16)	2.14E-09
PCK2	12.15	3.52E-30 (3.17E-27)	3.18E-09	NDUFB1	6.99	3.71E-11 (8.90E-09)	1.59E-09	TGFA	9.34	1.44E-18 (2.32E-16)	2.12E-09
NCK2	12.35	5.16E-31 (1.16E-27)	3.13E-09	CALML3	6.92	5.77E-11 (9.59E-09)	1.57E-09	FAS	9.59	2.32E-19 (8.71E-17)	2.06E-09

ESPL1	12.03	1.11E-29 (5.56E-27)	3.13E-09	ERBB4	7	3.56E-11 (8.90E-09)	1.49E-09	FZD8	9.39	1.03E-18 (2.10E-16)	1.85E-09
POLA1	11.96	2.28E-29 (6.40E-27)	3.08E-09	HSPA8	7.17	1.33E-11 (7.40E-09)	1.38E-09	TPO	9.32	1.75E-18 (2.55E-16)	1.71E-09
PPP2R1A	11.93	3.02E-29 (6.80E-27)	3.04E-09	HSPA1A	7.35	4.53E-12 (7.14E-09)	1.19E-09	LDHC	9.38	1.08E-18 (2.12E-16)	1.63E-09
MGST1	12	1.55E-29 (6.16E-27)	2.99E-09	MECOM	7.09	2.12E-11 (7.40E-09)	1.05E-09	CDK6	9.47	5.76E-19 (1.57E-16)	1.48E-09
FYN	11.81	9.50E-29 (1.69E-26)	2.98E-09	DMD	7.13	1.71E-11 (7.40E-09)	9.76E-10	NOS2	9.43	7.80E-19 (1.94E-16)	1.47E-09
GNG4	11.77	1.30E-28 (2.01E-26)	2.71E-09	DNAH6	6.99	3.76E-11 (8.90E-09)	9.42E-10	MGAT1	9.35	1.35E-18 (2.32E-16)	1.40E-09
PRKAA2	11.8	9.79E-29 (1.69E-26)	2.29E-09	CYP2C18	6.92	5.66E-11 (9.59E-09)	8.65E-10	CDC45	9.83	3.60E-20 (6.89E-17)	1.30E-09
PNPT1	11.9	3.92E-29 (7.66E-27)	1.57E-09	MCM2	6.91	5.96E-11 (9.59E-09)	8.05E-10	EFNA4	9.35	1.36E-18 (2.32E-16)	1.27E-09
CACNB4	11.94	2.57E-29 (6.61E-27)	1.52E-09	DNAH11	6.89	6.57E-11 (9.85E-09)	8.03E-10	LPL	9.67	1.26E-19 (6.89E-17)	1.02E-09
BIRC3	11.98	1.81E-29 (6.26E-27)	1.35E-09	RFC3	7.02	3.12E-11 (8.90E-09)	7.78E-10	MAN1B1	9.35	1.41E-18 (2.32E-16)	7.15E-10
PRKAA1	12.08	6.76E-30 (4.35E-27)	1.05E-09	GSTM3	7.08	2.32E-11 (7.46E-09)	7.70E-10	PRKAB2	9.4	9.26E-19 (1.98E-16)	5.07E-10

WIPF3	11.91	3.48E-29 (7.25E-27)	4.72E-10	AMD1	6.9	6.30E-11 (9.78E-09)	3.29E-10	SUFU	9.42	8.21E-19 (1.94E-16)	5.06E-10

Supplementary Table S5. Top 30 Predictive Pathways by Subgroup (Asthma Cohort)

Common Pathway	t-stat	p-val(FDR)	Importance	Male Pathway	t-stat	p-val(FDR)	Importance	Female Pathway	t-stat	p-val(FDR)	Importance
Nitrogen metabolism	13.48	7.34E-36 (3.62E-34)	3.78E-07	Neurotrophin signaling pathway	7.55	1.44E-12 (3.76E-11)	3.10E-07	Tryptophan metabolism	10.16	2.90E-21 (4.98E-20)	2.82E-07
Pancreatic cancer	13.33	3.19E-35 (5.25E-34)	3.76E-07	MAPK signaling pathway	7.5	1.94E-12 (3.76E-11)	2.00E-07	Activation of PRC2.2 by Ubiquitination of H2AK119 in Germline Genes	10.36	6.15E-22 (1.62E-20)	2.44E-07
Glycine, serine and threonine metabolism	13.66	1.15E-36 (2.95E-34)	3.61E-07	Disassembly of MCC	7.46	2.40E-12 (3.76E-11)	1.81E-07	PPAR signaling pathway	10.55	1.32E-22 (5.38E-21)	2.38E-07
Insulin signaling pathway	13.51	5.22E-36 (2.95E-34)	3.51E-07	RAB7 Regulation of Endosome Maturation	7.56	1.32E-12 (3.76E-11)	1.78E-07	JAK-STAT signaling pathway	10.34	7.18E-22 (1.67E-20)	2.36E-07
Pyrimidine metabolism	13.45	9.40E-36 (4.13E-34)	3.43E-07	Alanine, aspartate and glutamate metabolism	7.46	2.39E-12 (3.76E-11)	1.75E-07	DCE to DNA Adducts	10.5	1.93E-22 (6.95E-21)	2.24E-07

PPAR signaling pathway	13.27	5.77E-35 (7.88E-34)	3.21E-07	Cysteine and methionine metabolism	7.73	4.62E-13 (3.76E-11)	1.73E-07	HIV GP120 to CXCR4 GNAQ PLCB G CALCINEURIN	10.17	2.68E-21 (4.81E-20)	2.09E-07
Type II Interferon to JAK-STAT Signaling Pathway	13.56	3.24E-36 (2.95E-34)	3.11E-07	PDGF PDGFR Signaling Pathway	7.5	1.85E-12 (3.76E-11)	1.65E-07	MDA5 IRF7 3 Signaling Pathway	10.34	7.00E-22 (1.67E-20)	1.89E-07
B Cell Receptor Signaling Pathway	13.56	3.28E-36 (2.95E-34)	3.07E-07	Glycolysis	7.57	1.25E-12 (3.76E-11)	1.64E-07	GF RTK RAS PI3K Signaling Pathway	10.55	1.36E-22 (5.38E-21)	1.88E-07
GF RTK PI3K Signaling Pathway	13.36	2.38E-35 (5.02E-34)	3.06E-07	Histidine metabolism	7.53	1.57E-12 (3.76E-11)	1.58E-07	GPCR PI3K Signaling Pathway	10.58	1.01E-22 (5.38E-21)	1.88E-07
Autoimmune thyroid disease	13.43	1.27E-35 (4.40E-34)	3.05E-07	MGLUR5 CA2 Apoptotic Pathway	7.5	1.95E-12 (3.76E-11)	1.58E-07	Endocytosis	10.45	2.80E-22 (9.23E-21)	1.85E-07
LHCGR GNAS Signaling Pathway	13.37	2.28E-35 (5.02E-34)	2.93E-07	CXCR4-GNB-G-PLCB-PKC Signaling Pathway	7.56	1.35E-12 (3.76E-11)	1.50E-07	Fructose and mannose metabolism	10.09	4.85E-21 (6.39E-20)	1.78E-07
ITGA B FAK CAS Signaling Pathway	13.39	1.78E-35 (4.40E-34)	2.76E-07	TGF-beta signaling pathway	7.46	2.47E-12 (3.76E-11)	1.47E-07	Dilated cardiomyopathy	10.31	8.76E-22 (1.92E-20)	1.78E-07

TLR7/8/9 IRF5 Signaling Pathway	13.24	7.83E-35 (1.03E-33)	2.69E-07	Progesterone-mediated oocyte maturation	7.45	2.63E-12 (3.78E-11)	1.46E-07	Microtubule Nucleation	10.56	1.22E-22 (5.38E-21)	1.68E-07
SARS-CoV-2 NSP1 to Translation Initiation	13.34	2.94E-35 (5.19E-34)	2.66E-07	Other glycan degradation	7.46	2.35E-12 (3.76E-11)	1.43E-07	Mutation Caused Aberrant HTT to MGLUR5 CA2 Apoptotic Pathway	10.1	4.55E-21 (6.20E-20)	1.67E-07
DNA End Resection	13.39	1.75E-35 (4.40E-34)	2.59E-07	Mutation Caused Aberrant Abeta to AGE-RAGE Signaling Pathway	7.51	1.81E-12 (3.76E-11)	1.43E-07	CXCL12 CXCR4 PKC ERK Signaling Pathway	10.43	3.48E-22 (1.06E-20)	1.58E-07
N-glycan biosynthesis	13.4	1.71E-35 (4.40E-34)	2.46E-07	Fc epsilon RI signaling pathway	7.43	2.92E-12 (3.79E-11)	1.38E-07	Electron Transfer in Complex I	10.64	6.37E-23 (5.38E-21)	1.46E-07
Focal adhesion	13.44	1.11E-35 (4.39E-34)	2.44E-07	Biosynthesis of unsaturated fatty acids	7.58	1.14E-12 (3.76E-11)	1.37E-07	Core NER Reaction	10.1	4.55E-21 (6.20E-20)	1.44E-07
Mutation Caused Aberrant TDP43 to Electron Transfer in Complex I	13.54	3.98E-36 (2.95E-34)	2.44E-07	Escherichia EspG to Microtubule RHOA Destruction	7.47	2.29E-12 (3.76E-11)	1.30E-07	Small cell lung cancer	10.19	2.19E-21 (4.32E-20)	1.42E-07

Type I Interferon Signaling Pathway	13.4	1.60E-35 (4.40E-34)	2.43E-07	Endocytosis	7.43	2.91E-12 (3.79E-11)	1.25E-07	Cytokine JAK-STAT Signaling Pathway	10.36	5.96E-22 (1.62E-20)	1.41E-07
Linoleic acid metabolism	13.35	2.74E-35 (5.19E-34)	2.39E-07	Type I IFN Signaling Pathway	7.79	3.35E-13 (3.76E-11)	1.19E-07	Gene Silencing by Methylation of H3K27 and Ubiquitination of H2AK119	10.1	4.38E-21 (6.20E-20)	1.35E-07
GnRH GNRHR Signaling Pathway	13.55	3.43E-36 (2.95E-34)	2.39E-07	IL6 Family to JAK-STAT Signaling Pathway	7.49	2.03E-12 (3.76E-11)	1.12E-07	Chronic myeloid leukemia	10.59	9.64E-23 (5.38E-21)	1.33E-07
Aminoacyl-tRNA biosynthesis	13.39	1.74E-35 (4.40E-34)	2.38E-07	HCMV UL33 to GNAI AC PKA Signaling Pathway	7.44	2.68E-12 (3.78E-11)	1.10E-07	Tight junction	10.11	4.10E-21 (6.20E-20)	1.33E-07
Glioma	13.52	4.85E-36 (2.95E-34)	2.28E-07	Iron to Anterograde Axonal Transport	7.46	2.39E-12 (3.76E-11)	8.86E-08	Mutation Inactivated PTCH1 to Hedgehog Signaling Pathway	10.61	8.19E-23 (5.38E-21)	1.28E-07
Biosynthesis of unsaturated fatty acids	13.36	2.41E-35 (5.02E-34)	2.19E-07	Graft-versus-host disease	7.48	2.10E-12 (3.76E-11)	8.06E-08	ACTH-Cortisol Signaling Pathway	10.63	6.78E-23 (5.38E-21)	1.25E-07

Mutation Caused Aberrant SNCA to Anterograde Axonal Transport	13.27	5.79E-35 (7.88E-34)	1.97E-07	Systemic lupus erythematosus	7.51	1.81E-12 (3.76E-11)	8.03E-08	Bladder cancer	10.15	3.10E-21 (5.10E-20)	1.24E-07
Ether lipid metabolism	13.28	5.43E-35 (7.88E-34)	1.89E-07	NOTCH Overexpression to Transcriptional Activation	7.78	3.55E-13 (3.76E-11)	7.64E-08	Glycosaminoglycan biosynthesis heparan sulfate	10.66	5.52E-23 (5.38E-21)	1.21E-07
TLR7 9 IRF7 Signaling Pathway	13.34	3.02E-35 (5.19E-34)	1.84E-07	Tryptophan metabolism	7.64	8.35E-13 (3.76E-11)	7.02E-08	SARS-CoV-2 NSP1 to Translation Initiation	10.62	7.35E-23 (5.38E-21)	1.12E-07
CXCL12 CXCR4 Signaling Pathway	13.34	2.95E-35 (5.19E-34)	1.68E-07	Beta-Alanine metabolism	7.48	2.17E-12 (3.76E-11)	6.71E-08	CXCR4 GNAQ PLCB G CALCINEURIN Signaling Pathway	10.14	3.28E-21 (5.19E-20)	1.10E-07
IGH MMSET Fusion to Transcriptional Activation	13.28	5.73E-35 (7.88E-34)	1.23E-07	Mutation Inactivated ATP2B3 to Angiotensin II Signaling Pathway	7.63	8.72E-13 (3.76E-11)	6.66E-08	Long-term depression	10.17	2.56E-21 (4.81E-20)	1.05E-07
Scrapie Conformation PrP Sc to ER Stress	13.31	4.09E-35 (6.46E-34)	1.14E-07	TCR PLCG ITPR Signaling Pathway	7.51	1.78E-12 (3.76E-11)	5.52E-08	DNA Replication Licensing	10.21	1.90E-21 (3.95E-20)	8.53E-08
